# Stringent control of the RNA-dependent RNA polymerase translocation revealed by multiple intermediate structures

**DOI:** 10.1038/s41467-020-16234-4

**Published:** 2020-05-25

**Authors:** Meihua Wang, Rui Li, Bo Shu, Xuping Jing, Han-Qing Ye, Peng Gong

**Affiliations:** 1 Key Laboratory of Special Pathogens and Biosafety, Wuhan Institute of Virology, Center for Biosafety Mega-Science, Chinese Academy of Sciences, No.44 Xiao Hong Shan, Wuhan, Hubei 430071 China; 2 University of Chinese Academy of Sciences, Beijing, 100049 China; 3 Drug Discovery Center for Infectious Diseases, Nankai University, Tianjin, 300350 China

**Keywords:** Enzyme mechanisms, Virology, X-ray crystallography

## Abstract

Each polymerase nucleotide addition cycle is associated with two primary conformational changes of the catalytic complex: the pre-chemistry active site closure and post-chemistry translocation. While active site closure is well interpreted by numerous crystallographic snapshots, translocation intermediates are rarely captured. Here we report three types of intermediate structures in an RNA-dependent RNA polymerase (RdRP). The first two types, captured in forward and reverse translocation events, both highlight the role of RdRP-unique motif G in restricting the RNA template movement, corresponding to the rate-limiting step in translocation. By mutating two critical residues in motif G, we obtain the third type of intermediates that may mimic the transition state of this rate-limiting step, demonstrating a previously unidentified movement of the template strand. We propose that a similar strategy may be utilized by other classes of nucleic acid polymerases to ensure templating nucleotide positioning for efficient catalysis through restricting interactions with template RNA.

## Abstract (web summary)

During translocation by viral RNA-dependent RNA polymerases (RdRP), the template-product RNA duplex moves in an asymmetric manner. Here the authors describe several crystal structures of RdRP translocations intermediates that, along with enzymological data, provide a comprehensive view of RdRP translocation.

## Introduction

Processive nucleic acid polymerases are molecular machines that carried out the template-directed biosynthesis of long DNA and RNA chains using nucleoside triphosphates (NTPs) as substrates, playing essential roles in nearly all life forms. These polymerases are capable of forming an elongation complex (EC) that processively carries out thousands of nucleotide addition cycles (NACs), and the mechanism by which the EC utilizes to execute each NAC is essential to the understandings of these enzymes. An NAC contains at least four micro-steps: the template-directed binding of NTP at or near the polymerase active site that is catalytically “open”, the active site closure defined by conformational changes of key polymerase motifs for optimal catalysis, the formation of the phosphodiester bond (i.e., chemistry), and the translocation event that moves the polymerase one register in the downstream direction of the nucleic acid template^1–3^.

For more than two decades, insights into the polymerase catalysis have been benefitted from numerous three-dimensional structures defining distinct states of the polymerase in the NAC. To date, the visually most significant conformational changes between different NAC states are mainly associated with the active site closure step. Relatively large-scale conformational changes were observed in A-family (also known as Pol I family) polymerases and multi-subunit DNA-dependent RNA polymerases^4–7^. For instance, the fingers domain of bacteriophage T7 RNA polymerase undergoes a dramatic rotational movement to allow the O-helix to reposition the NTP from a “pre-insertion site” to the catalytic competent insertion site^4,5^, and the trigger loop of yeast RNA polymerase II (pol II) becomes more ordered and moves to the proximity of the nucleotide addition site (“A” site, and equivalent to “insertion site”) upon active site closure to allow direct and indirect interactions with all moieties of NTP^7^. A different mode of polymerase active site closure is represented by viral RNA-dependent RNA polymerases (RdRPs). Viral RdRP comprises palm, fingers, and thumb domains as with other single-subunit polymerases, but forms a unique encircled architecture through fingers-thumb interactions that is absent in A-family polymerases^8^. The structural rearrangements leading to the active site closure in viral RdRPs are mostly limited to their palm domain with the hallmark being a small-scale inward movement of the catalytic motifs A/D backbone to facilitate catalysis^9^.

Translocation is generally regarded as the most important post-chemistry NAC event. It changes the whole footprint of polymerase on the nucleic acids by one register and moves the newly incorporated nucleotide out of the active site, thus being a possible and interesting target for polymerase intervention independent of the pre-chemistry NTP substrate selection. In A-family polymerases, translocation is facilitated by the O-helix movement that is essentially the reversion of the motion required for active site closure. This conformational change allows the side chain of an aromatic residue (e.g., Y631 in T7 RNA polymerase) to “push” the nascent base pair from the +1 site to the −1 site^1,5,6^. An analogous mechanism was proposed in multi-subunit RNA polymerases that the bending of bridge helix may account for the “push” based on a straight bridge helix observed in pre-translocation yeast pol II ECs and a bent bridge helix in apo bacterial RNA polymerases^10,11^. However, direct evidence of a bent bridge helix “pushes” against the nascent base pair in an intermediate EC is necessary to prove this hypothesis. It is conceivable that polymerases utilize key parts in the vicinity of the nascent base pair to facilitate translocation after catalysis. However, the mode of the nucleic acids movement, in particular, that of the template-product duplex relative to the polymerase active site during translocation remains largely elusive. In numerous polymerase EC structures, the duplex upstream of the active site is either in pre- or in post-translocation states. Whether stable translocation intermediates between the pre- and post-translocation states exist, or could such intermediates be captured by structural biology approaches to interpret translocation mechanism, remains to be clarified.

We previously developed a methodology in efficiently crystallizing picornavirus RdRP EC by taking advantage of RNA-mediated crystal contacts^12^. This approach allowed us to efficiently obtain versatile crystal forms of RdRP ECs, and the variations in lattice-induced restraints on the function of polymerase provided potentials to stabilize or trap low-energy intermediates that may be short-lived in solution. Building on our recent work reporting an enterovirus 71 (EV71) RdRP EC with an intermediate conformation of its template-product duplex^13^, here we report three types of translocation intermediate crystal structures that define multiple nucleic acid conformations bridging the pre- and post-translocation states, both in the regulate forward translocation and in an unusual reverse translocation induced by pyrophosphate (PP_i_) and divalent metal ions. These structural data, in combination with enzymology characterization of the RdRP, highlight the key role of RdRP motif G in controlling the critical movement of the template RNA strand that is probably the rate-limiting step for both forward and reverse translocations. Our work provides important mechanistic details towards the understanding of how translocation occurs in viral RdRPs and will serve as an important reference for understanding the nucleic acid polymerase NAC in general.

## Results

### A translocation intermediate with a mixture of two states

**Fig. 1:**
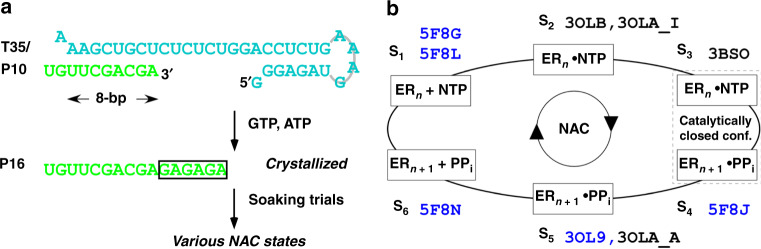
A schematic diagram for EV71 RdRP EC assembly, crystallization, and crystal soaking and a six-state model for the viral RdRP NAC. **a** The EC was assembled using the T35/P10 construct upon addition of a G-A-G-A-G-A hexa-nucleotide. The 16-mer (P16)-containing EC was crystallized and subjected to different soaking trials to capture various NAC states. **b** The previously proposed six-state RdRP NAC starts with the state 1 (S_1_) complex with an empty and open conformation active site, and the NTP binding leads to the state 2 (S_2_) complex that remains in the open conformation. In most cases, the cognate NTP induces a conformational change to close the active site (corresponding to the active site closure) to reach state 3 (S_3_), and then completion of the phosphoryl transfer indicates the formation of the state 4 (S_4_) complex that remains in the closed conformation. The active site often resets to open conformation before translocation and the corresponding complex was assigned as the state 5 (S_5_) complex. The state 6 (S_6_) complex represents any intermediates that poised between the pre- and post-translocation states. The representative PDB entries are provided next to the state symbols and reference entries used to help present the intermediate structures in this study are shown in blue.

**Fig. 2:**
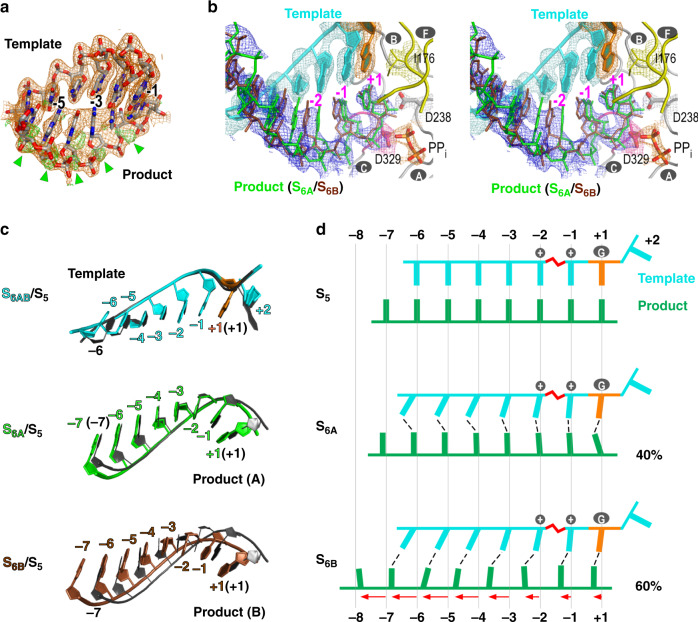
A structure of RdRP translocation intermediate contains two alternative conformations of the product RNA. **a** Electron density maps (orange: 2F_o_-F_c_ contoured at 1.3 σ; green: F_o_-F_c_ contoured at 3.5 σ) with a pre-translocation state model overlaid suggest alternative conformations for the product strand. Green triangles indicate an array of phosphate density inconsistent with the model. **b** Stereo-pair images of the intermediate structure S_6A_/S_6B_ with a composite SA omit electron density map (contoured at 0.7 σ) overlaid. Coloring scheme: template in cyan (+1 nucleotide in orange), S_6A_ product in green, S_6B_ product in brown, palm in gray (YGDD sequence in magenta), ring finger subdomain in yellow, and PP_i_ in orange. Capital letters with gray background indicate corresponding RdRP motifs. The side chains of several conserved residues, I176 of motif F, D238 of motif A, and D329 of motif C, are shown as sticks. **c** RNA-only comparison of the translocation intermediate (color) and the pre-translocation state (dark gray, PDB entry 3OL9, chain A complex). **d** Schematic illustration of the RNA movement suggested by the intermediate structure. Vertical lines indicate reference nucleotide positions. Zigzagged red symbol indicates the unusual backbone conformation of the template −2 position. The dashed lines indicate baseparing interactions. Capital letter G indicates motif G hurdle residues. The “+” symbols indicate the basic residues K127/R188 that can interact with the -1/-2 template phosphates, respectively. The red arrows indicate the distance and direction of backbone movement of the product nucleotides. Forty and 60% denote the crystallographic occupancy of S_6A_ and S_6B_, respectively.

Building on the previously established methods in assembling enterovirus RdRP EC and in capturing various EC states^12,13^, we used an EV71 RdRP bearing a C291M mutation in such approaches, since the equivalent PV RdRP mutant (C290M) was shown to produce higher quality EC crystals (Fig. 1a)^12^. The six-state nomenclature of the RdRP catalytic cycle suggested in our previous work was used in this study to aid the illustration of translocation intermediates and the mechanisms suggested by them (Fig. 1b)^3,9,14^. Besides capturing a state 6 (S_6_) translocation intermediate after incorporation of a C-C-U tri-nucleotide with almost identical conformation to the previously reported intermediate (pdb entry 5F8N, Supplementary Fig. 1), we observed unusual electron densities for some of the EC structures derived from similar crystal soaking strategies (Fig. 2 and Table 1, S_6A_/S_6B_ (C_3_) structure, 2.3 Å resolution, C_3_ denotes the third NAC). While the template strand density of this type of structure appeared normal, the density of the product strand clearly indicated the presence of two sets of backbone phosphates. When a pre-translocation product RNA was modeled, strong positive density appeared between the modeled phosphates and continuous density was observed for an array of neighboring product bases (Fig. 2a). By modeling two alternate conformations of the product strand, this unusual density can be readily interpreted (Fig. 2b–d). Accordingly to the six-state nomenclature of the RdRP NAC suggested in our previous work with S_6_ representing translocation intermediates, we assign these two EC states as S_6A_ and S_6B_. While both states share the same template model that is nearly identical to that in the previously reported S_6_ structure, the two product models differ in the extent of translocation with S_6B_ leading S_6A_ with an occupancy of 60% and 40%, respectively (Fig. 2d). In order to better visualize the intermediate states, here we use previously reported representative state 1 (S_1_, post-translocation with empty active site) and state 5 (S_5_, pre-translocation with active site reset to open conformation) structures as the primary references (S_1_: 5F8G/5F8L; S_5_: 3OL9, chain A)^9,13^, and several conserved residues, including I176 in motif F, D238 in motif A, and D329 in motif C, were used to aid structural comparisons (Fig. 2b). Comparing to the S_5_ product RNA, the S_6A_ product slightly moved toward the upstream as an entity, while the S_6B_ product had the upstream nucleotides move by longer distances than the downstream nucleotides and is nearly identical to the previously reported S_6_ product (Fig. 2c–d; Supplementary Movie 1: the first clip shows the modeled transition from S_5_ to S_6_). We have proposed that these intermediates can form fast equilibrium with the pre-translocation state, and the observation of the S_6A_ and S_6B_ co-existence suggests that these intermediates are indeed stable enough to form distributed states and to be captured by crystallography.

### A PPi-induced reverse translocation intermediate structure

**Fig. 3:**
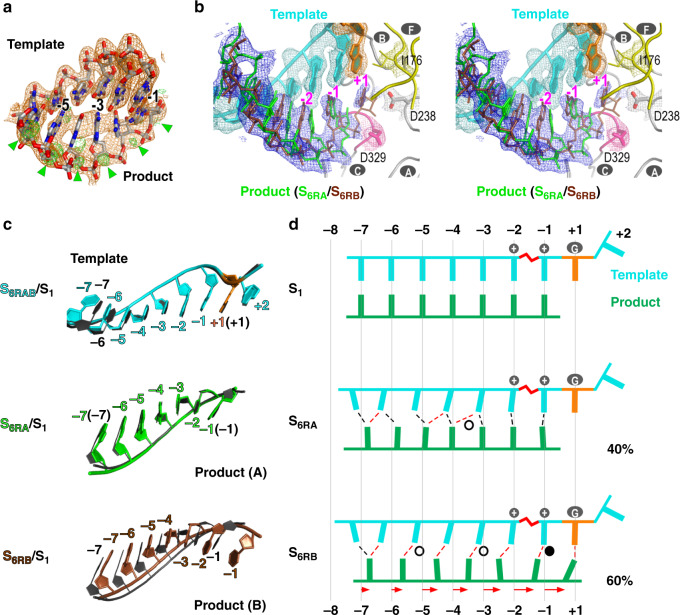
A structure of RdRP reverse translocation intermediate contains two alternative conformations of the product RNA. **a** Electron density maps (orange: 2F_o_-F_c_ contoured at 1.8 σ; green: F_o_-F_c_ contoured at 3.5 σ) with a post-translocation state model overlaid suggest alternative conformations for the product strand. Green triangles indicate an array of phosphate density inconsistent with the model. **b** Stereo-pair images of the intermediate structure S_6RA_/S_6RB_ with a composite SA omit electron density map (contoured at 0.8 σ) overlaid. Capital letters with gray background indicate corresponding RdRP motifs. The nucleotide position was labeled according to the template strand. **c** RNA-only comparison of the translocation intermediate (color) and the post-translocation reference state (dark gray, PDB entry 5F8L). **d** Schematic illustration of the RNA movement suggested by the reverse translocation intermediate structure. Labeling, symbols, and coloring scheme are the same as in Fig. 2, with the red dashed lines indicating newly formed baseparing interactions, empty and solid circles indicating regular and wobble base pairs, respectively.

The observation of relative high level of freedom during translocation for the product strand intrigued us to test whether such a phenomenon is also applied to translocation in the reverse direction, a process starting from the post-translocation state to the pre-translocation state in the previous cycle. Although not previously observed in nucleic acid polymerases, such in-crystal reverse translocation may possibly occur as this EC lattice was shown to accommodate multiple rounds of catalysis and translocation^13^. By soaking native EC crystals in CTP-containing solutions, we were able to allow the polymerase to reach the state 1 of the third cycle (C_3_S_1_: after two incorporation events and two translocation events) (Supplementary Fig. 2, top left). Subsequently, these S_1_ EC crystals were soaked in solution containing PP_i_ and MnCl_2_. In a time scanning experimental setting, applying long soaking time resulted in completion of the reverse translocation and the EC had “walked” back to a pre-translocation state of the previous cycle (C_2_S_4/5_, Supplementary Fig. 2, bottom right), while shorter soaking time led to an intermediate state again with strong indication of a mixed distribution of product strand phosphates and continuous electron density for neighboring bases (Supplementary Fig. 2, bottom left; Fig. 3a and Table 1, S_6RA_/S_6RB_ (C_2_) structure, where “R” denotes reverse, 2.2 Å resolution). In contrast, the entire backbone of the template strand stayed largely at the post-translocation state and the density of bases were localized. Alternate conformation modeling and refinement indicated that two populations of the product strand existed with different reverse translocation extents (Fig. 3b–d). The first population represents an intermediate state with small-scale reverse translocation of a few upstream product nucleotides (positions −5 to −7) (state 6_RA_ or S_6RA_, 40% occupancy), while the second population had the entire product backbone moved toward the downstream with an average distance about one-half register and having the downstream nucleotides moved farther than the upstream ones (Fig. 3c–d, S_6RB_, 60% occupancy; Supplementary Movie 2: the clip shows the modeled transition from S_1_ to S_6RB_). Unlike what was observed in the forward translocation intermediates, the majority of the original base pairs in the S_6RB_ were no longer maintained. Slippage occurred between the two RNA strands, resulting in a mixture of mismatches, G:U wobble base pairs, and a G:C base pair (Fig. 3d and Supplementary Movie 2). When MgCl_2_ was used in reverse translocation soaking trials, an intermediate structure can also be captured with consistent features of the MnCl_2_-derived S_6RA_/S_6RB_ structure (Supplementary Fig. 2, top right structure). It seems that the observed heterogeneity of the product strand conformation in both forward and reverse translocation is relatively independent, as not only the template strand, but also the product-interacting RdRP regions have defined electron density to indicate conformation homogeneity (Figs. 2b, 3b, and Supplementary Fig. 3).

**Table 1:** X-ray diffraction data collection and structure refinement statistics.

	6LSE: S_6A_/S_6B_ (C_3_)	6LSF: S_6RA_/S_6RB_ (C_3_)	6LSG: S_6M_ (C_0_)	6LSH: S_6M_ (C_2_)
Data collection^a^				
Space group	P2_1_2_1_2_1_	P2_1_2_1_2_1_	P2_1_2_1_2_1_	P2_1_2_1_2_1_
Cell dimensions				
a, b, c (Å)	62.6, 77.0, 156.7	61.4, 76.4, 155.0	63.6, 77.2, 154.2	62.9, 77.4, 155.8
α, β, γ (◦)	90, 90, 90	90, 90, 90	90, 90, 90	90, 90, 90
Resolution (Å)^b^	50.00–2.25 (2.33–2.25)	50.00–2.15 (2.23–2.15)	50.00–2.12 (2.20–2.12)	50.00-2.23 (2.31-2.23)
No. unique reflections	32,014	36,176	42,624	35,885
*R*_merge_	0.082 (0.52)	0.061 (0.34)	0.029 (0.16)	0.036 (0.42)
*R*_meas_	0.092 (0.60)	0.076 (0.47)	0.034 (0.23)	0.049 (0.58)
CC_1/2_	0.992 (0.77)	0.999 (0.88)	0.999 (0.98)	1.002 (0.84)
I/σI	13.7 (2.1)	16.8 (2.6)	44.6 (6.9)	31.9 (2.5)
Completeness (%)	87.2 (75.2)	89.5 (66.8)	96.9 (69.4)	95.2 (79.1)
Redundancy	4.4 (3.6)	4.7 (3.7)	6.2 (4.5)	5.5 (3.9)
Structure refinement				
Resolution (Å)	2.25	2.15	2.12	2.23
*R*_work_/*R*_free_^c^ (%)	19.9/24.0	21.5/25.1	19.0/21.7	20.8/24.5
No. atoms				
Protein/RNA	3616/340	3629/405	3,638/386	3,617/405
Ligand/Ion/Water	19/1/170	5/1/92	5/1/228	5/1/103
B-factors (Å^2^)				
Protein/RNA	45.7/49.3	53.1/65.0	43.4/54.7	54.4/74.0
Ligand/Ion/Water	56.2/32.5/44.8	48.4/44.6/49.6	37.9/36.3/44.4	48.1/45.7/50.9
RMSD				
Bond lengths (Å)	0.007	0.008	0.007	0.008
Bond angles (◦)	0.851	0.9	0.833	0.851

^a^One crystal was used for data collection for each structure.

^b^Values in parentheses are for the highest-resolution shell.

^c^5% of data are taken for the *R*_free_ set, and the same *R*_free_ set is applied for the all structures.

While both the translocation directions (in particular the movement of the product strand) and the interaction details between the two RNA strands are different for forward and reverse translocations, as suggested by the captured intermediates (comparing Figs. 2d and 3d). A striking common feature for both modes of translocation is that the template backbone, in particular for positions −2 to +1, stayed fixed relative to its starting point of translocation. As we pointed out previously^12,13^, this phenomenon is probably related to two motif G residues (T114-S115 in EV71 RdRP, “G” symbol in Figs. 2d and 3d) that create a hurdle-like steric blockage right at the +1/+2 junction of the template backbone. Aside from this, the −1 and −2 template phosphates interact with two residues with basic side chains (“+” symbols in Figs. 2d and 3d), K127 and R188, help maintain an unusual backbone conformation of the −2 template and may play an auxiliary role to control the template backbone movement during translocation^12^, as both K127 and R188 are not conserved in viral RdRPs. In order to complete either forward or reverse translocation, the template +1 position phosphate needs to “leap” over the hurdle, and overcoming such an energy barrier is probably the rate-limiting step during translocation.

### The hurdle residues in RdRP motif G are tunable

**Fig. 4:**
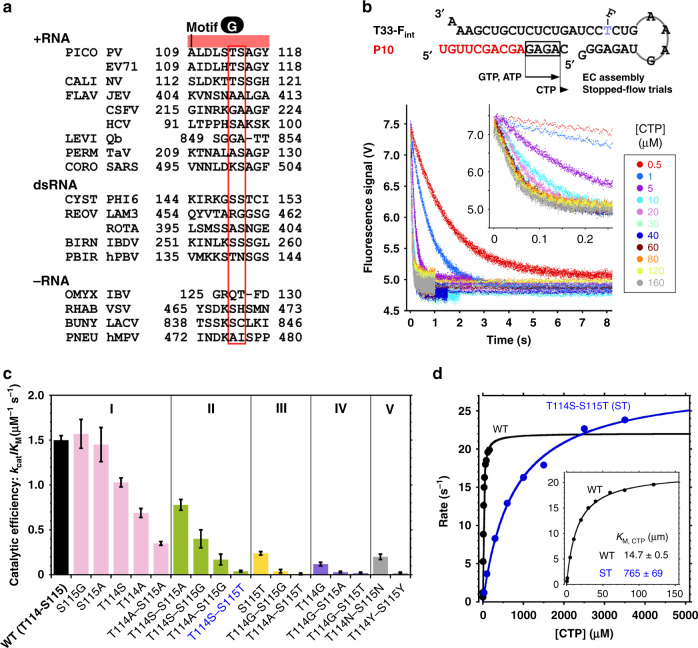
Enzymology characterization of EV71 RdRP and its mutants bearing mutation(s) in the motif G hurdle residues. **a** A structure-based sequence alignment of RdRP motif G. Representative RdRPs from positive-strand (+RNA), double-stranded (dsRNA), and negative-strand (-RNA) RNA viruses were chosen for comparison (PICO/PV, EV71: *Picornaviridae*/poliovirus, enterovirus 71; CALI/NV: *Caliciviridae*/norovirus; FLAV/JEV, CSFV, HCV: *Flaviviridae*/Japanese encephalitis, classical swine fever, hepatitis C viruses; LEVI/Qb: *Leviviridae*/bacteriophage Qβ; PERM/TaV: *Permutotetraviridae*/thosea asigna virus; CORO/SARS: *Coronaviridae*/severe acute respiratory syndrome coronavirus; CYST/PHI6: *Cystoviridae*/bacteriophage ϕ6; REOV/LAM3, ROTA: *Reoviridae*/reovirus λ3, rotavirus; BIRN/IBDV: *Birnaviridae*/infectious bursal disease virus; PBIR/hPBV: *Picobirnaviridae*/human picobirnavirus; OMYX/IBV: *Orthomyxoviridae*/influenza B virus; RHAB/VSV: *Rhabdoviridae*/vesicular stomatitis virus; BUNY/LACV: *Peribunyaviridae*/La Crosse bunyavirus; PNEU/hMPV: *Pneumoviridae*/human metapneumovirus). Hurdle residues are highlighted by the red box. **b** The sequences of the T33-F_int_/P10 construct used in the stopped-flow assay and the data from the WT construct showing faster fluorescence signal decrease with higher CTP concentration. F_int_ denotes a fluorescein-labeled nucleotide at an internal position. When GTP and ATP were provided as the only NTP substrates, the 10-mer primer (P10) was extended to a 14-mer product for EC assembly (the incorporated G-A-G-A tetra-nucleotide is boxed). The assembled EC was then used in stopped-flow trials by mixing with CTP for monitoring the single-nucleotide conversion from the 14-mer to a 15-mer. **c** A comparison of catalytic efficiency for WT and hurdle residue mutants. All constructs were classified into five categories based on their kinetics behaviors (Supplementary Fig. 4). The error bars indicate propagated fitting errors (Supplementary Table 1). **d** Michaelis–Menten curve fitting of the WT and ST mutant data. *K*_M_ values with fitting errors were indicated.

Possibly due to low sequence conservation, the function of motif G has not been well clarified since its discovery as an RdRP-unique motif (Fig. 4a)^15^. The EV71 RdRP G117-equivalent residue exhibits relatively high-level conservation in the positive-strand  and double-stranded (ds) RNA viruses. This residue does not interact with the RNA and the preference of a glycine could be related to its involvement in the folding of a sharp turn (corresponding to residues 117–120 in EV71 RdRP), thus only playing a structural role. Among the motif G residues, the aforementioned hurdle residues are of particular interest due to their critical spatial position and their roles in restricting the template RNA movement as suggested by both forward and reverse translocation intermediate structures. With an aim to dissect the role of the motif G hurdle residues in translocation and in RdRP NAC in general, we compared representative RdRP structures to help locate structurally equivalent motif G residues and found that the majority of residues at these two positions are serine (S), alanine (A), threonine (T), and glycine (G) (Fig. 4a). This observation suggests that RdRPs tend to use small-side-chain residues at these two positions. The low-level sequence conservation and high-level conservation in side chain size intrigued us to screen for motif G mutants that could possibly change the nature in restricting the movement of the template strand while keeping the ability in catalysis. By applying all possible combination of S/A/T/G residues at RdRP positions 114 and 115, we made 15 mutants for a comparison with the WT (T114-S115). We also made four mutants with identical residues at both positions for comparison, and medium-side-chain residues asparagine (N), leucine (L) and large-side-chain residues phenylalanine (F), tyrosine (Y) were chosen. Except for the double L and double F mutants (Supplementary Table 1), these constructs were able to form ECs after incorporating a G-A-G-A tetra-nucleotide directed by a “CUCU” template sequence (Fig. 4b, top), allowing us to determine the catalytic efficiency (denoted by *k*_cat_/*K*_M,NTP_) in a stopped-flow fluorescence-based single nucleotide incorporation assay modified from previously reported methods (Fig. 4b–d)^16^. For the WT construct and five mutants (S115G, S115A, T114S, T114A, and T114A-S115A), the fluorescence signal change upon CTP addition fits well to a single-exponential decay model, while the signal change for other mutants deviated from the WT-model to different extents, possibly indicating conformational alteration brought by some of the mutants. We therefore classified all 18 constructs in five classes (I-V) based on the observed kinetics behaviors (Fig. 4c and Supplementary Fig. 4). Nevertheless, the same fitting strategy was applied for all constructs to determine the *k*_cat_ and *K*_M,NTP_ values (Table S1). It turned out that more than half of the mutants had at least 10% catalytic efficiency of the WT-level (11–105%), to some extent supporting the postulation that residues at these two positions may be variable to some extent while maintaining the catalytic capability.

### An “ST” mutant leads to a transition-state-like structure

**Fig. 5:**
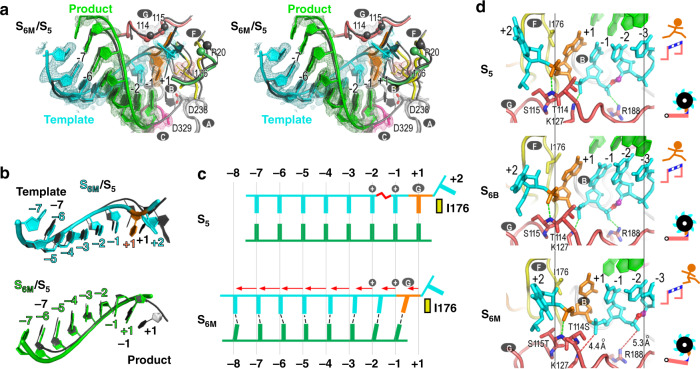
A structure of translocation intermediate exhibiting critical movement of the template RNA in an RdRP bearing mutations in the motif G hurdle residues. **a** Stereo-pair images of the intermediate structure S_6M_ with a composite SA omit electron density map (contoured at 1.2 σ) overlaid. **b** RNA-only comparison of this translocation intermediate (color) and the pre-translocation reference state (dark gray, PDB entry 3OL9). **c** Schematic illustration of the RNA movement suggested by the intermediate structure. Labeling, symbols, and coloring scheme are the same as in Fig. 2. For the S_6M_ structure, motif G hurdle residues and motif F I176 moved with the template strand accordingly, while the electrostatic interactions between -1/-2 template phosphates and K127 and R188 were lost and the unusual -2 template backbone conformation reset to normal. **d** A structural comparison of pre-translocation (S_5_), early (S_6B_), and late (S_6M_) translocation intermediates states highlighting the coordinative conformational changes of the template strand and key residues in motifs F and G. The athlete-hurdle and ratcheting wheel cartoons are provided for better illustration of critical role of the hurdle residues 114–115. Labeling, symbols, and coloring scheme are the same as in Fig. 2 with motif G in pink.

For all five classes of mutants, the first three exhibited relative efficient EC assembly. We next tested one mutant from each class for EC crystallization to see whether the mutation could change the behavior in template strand restriction during translocation. Among these mutants, a class II mutant T114S-S115T (abbreviated as ST) yielded EC crystals that are suitable for structure determination. This mutant has a *k*_cat_ value comparable to the WT but its *K*_M,NTP_ is about 50-fold of the WT level (Fig. 4c–d). The much lower NTP affinity could indicate the alteration of the catalytic pocket at the NTP binding stage. Interestingly, the ST mutant-derived EC structure indeed exhibited a previously unidentified conformation with the −2 to +2 template nucleotides clearly occupying intermediate positions, while the product strand almost reached the post-translocated position (Table 1 and Fig. 5, S_6M_ (C_0_) structure, 2.1 Å resolution, C_0_ indicates that the intermediate state is in an NAC prior to the corresponding post-translocation state of this native EC). Although the nearly full-register movement of product strand had vacated the active site, we did not observe NTP-like electron densities in it, and therefore the incoming NTP-assisted translocation is not evident. Previously determined native enterovirus EC crystal structures all adopted the post-translocation conformation, and therefore it is probable that the hurdle residue mutation relatively destabilize the post-translocation state, allowing the capture of the observed intermediate state. Comparing to the pre-translocation state, the template +1 phosphate moved by 2.2 Å toward the upstream and residue 115 backbone amide nitrogen interacting with the phosphate oxygen also moved 1.7 Å accordingly (Fig. 5d). The electrostatic interactions between the K127/R188 side chains and the −1/−2 template phosphates were lost, and the unusual backbone conformation switched to regular conformation (Fig. 5d and Supplementary Fig. 5, compare S_5_ and S_6M_). Therefore, the S_6M_ represents a late stage in the translocation process and probably mimics the actual transition state of the rate-limiting step in forward translocation for an RdRP with a WT-sequence in motif G (Supplementary Movie 1: the second clip shows the modeled transition from S_6_ to S_6M_ and the third clip shows the modeled transition from S_6M_ to S_1_ of the next cycle). The capture of an intermediate state in the native EC crystal suggests that the stability of the observed intermediate is higher or at least comparable to the pre- and post-translocation states for the ST mutant. We therefore tested NTP soaking experiments for the ST mutant-derived EC crystals to see whether a pre- or post-translocation state can be captured upon catalysis and translocation. After incorporating a C–C dinucleotide upon CTP soaking, the EC structure exhibited an almost identical S_6M_ intermediate state (Table 1, S6M (C_2_) structure, 2.2 Å resolution, and Supplementary Fig. 6), indicating that the energy landscape for the translocation process may have been indeed changed by the ST-mutation.

## Discussion

The biosynthesis of long chain nucleic acid is essential for most forms of life and comprises thousands of NACs carried out by different types of processive nucleic acid polymerases. Catalysis and translocation need to be well coupled within each NAC and between neigboring NACs to fulfil rapid synthesis in a processive manner (tens to hundreds of nucleotides per second). However, these two processes have distinct characters and requirements. For catalysis, the polymerase needs to precisely position the +1 template nucleotide, ensuring the NTP recognition and subsequent conformational changes to close the active site for the phosphoryl transfer reaction. The conformational changes for all types of polymerases, in an induced-fit manner, unexceptionally occurred around the NTP substrate^4–7,9,17^. Therefore, the polymerase is relatively “fixed” on the nucleic acid in the catalysis part of the NAC. Once chemistry occurs, the goal of translocation is to “move” the entire polymerase one-register downstream and therefore to set up the stage for the next catalysis event in the next NAC. Hence, the RNA-RdRP interaction changes brought by the global movement of RNA relative to the RdRP during translocation are not expected to be localized around the active site, and the relative movement between the polymerase and nucleic acids and the components that control or contribute to these movements are essential to the understanding of translocation. However, as detailed below, only small-scale conformational changes around the active site of the RdRP occur during translocation as the RNA “threads” through the polymerase. Although the detailed translocation mechanism suggested by the intermediate structures reported here may only applicable for viral RdRPs, other classes of polymerases may utilize similar strategies to control translocation in accordance with catalysis.

**Fig. 6:**
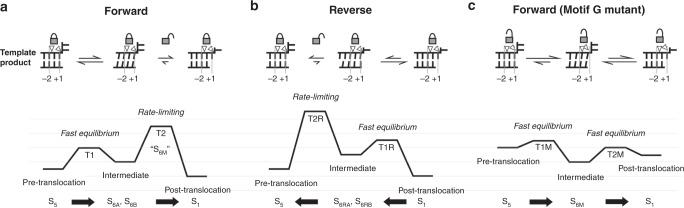
Schematic free energy diagrams for working models depicting RdRP translocations. **a** Forward translocation. The pre-translocation state (S_5_) could establish fast equilibrium with the intermediate states (S_6A_/S_6B_), and the subsequent transition to the post-translocation state (S_1_) is rate-limiting. **b** Reverse translocation. The post-translocation state could establish fast equilibrium with the intermediate states (S_6RA_/S_6RB_), and the subsequent transition to the pre-translocation state (S_5_) is rate-limiting. **c** Forward translocation for the ST mutant. Comparing to the model in panel a, the intermediate state (S_6M_) becomes the most stable species but the free energy differences between the intermediate and the pre- and post-translocation states (S_5_ and S_1_) and the corresponding energy barriers are lower. The S_6M_ in this panel mimics the T2 transition state in panel a (shown as “S_6M_”). Empty triangles indicate the motif G hurdle residues (right) and the −1/−2 phosphates-interacting basic residues (left) that “lock” the template (Fig. 5d). These locking interactions only unlock at the rate-limiting steps in models in (**a**–**b**), while in the panel c model, the template locking is overall weakened (partially locked symbols) by the ST mutation for all states. T1/T2, T1R/T2R, T1M/T2M are proposed transition states for forward, reverse, ST-mutant-derived translocations. The horizontal bars are only used to indicate relative free energy levels proposed in our working model and do not indicate quantitative suggestions.

The observation of the three types of translocation intermediates allows a detailed analysis of RdRP translocation mechanism in the context of previously proposed models. The Brownian ratchet model emphasizes the fast equilibrium between the pre- and post-translocation states and the PP_i_ or incoming NTP as the “ratchet” for stabilizing the pre- and post-translocation states, respectively^18,19^. In forward and reverse translocations, fast equilibrium may be established between the starting state of each translocation event and the structurally observed intermediates (Fig. 6, a-b: S_5_ and S_6A_/S_6B_; S_1_ and S_6RA_/S_6RB_), and the observation of overall longer moving distances for the leading nucleotides (i.e., upstream nucleotides in Fig. 2d and downstream nucleotides in Fig. 3d) in these intermediates is also consistent with thermo-fluctuation feature of the Brownian motion (Supplementary Movie 1, the first clip; Supplementary Movie 2). However, the motif G hurdle residues hinder these intermediates from easily reaching the end point of the corresponding translocation event (S_1_ and S_5_ for forward and reverse translocations, respectively), making the entire translocation event inconsistent with the pure Brownian ratchet mechanism. The power stroke model features a conformational change to convert the pre- to post- translocation states in a single and largely irreversible step. This model was proposed in T7 RNA polymerase (a member of the A-family polymerases) mainly due to the structural observation of the aforementioned polymerase component “O-helix” sitting at an ideal position after active site closure^5^. Presumably, the reverse conformational change (comparing to that occurs in the active site closure) of the O-helix allows a tyrosine (Y639) to move toward the +1 template-product base pair by 3.4 Å, thereby “pushing” the DNA-RNA heteroduplex to the post-translocational position. The relative “irreversible” feature of the power stroke mechanism fits well with the rate-limiting steps of both forward and reverse translocation processes, at which a conformational change is required to release the locking interactions between the motif G hurdle residues and the template strand backbone (Fig. 6a–b; Supplementary Movie 1, the combination of the second and third clips shows the modeled process in releasing the locking interactions in forward translocation). The S_6M_ structure further suggests that mutating the motif G hurdle residues could alter the relative stability of key states in translocation, presumably through weakening of the locking interactions (Fig. 6c). For the ST mutant, rate-limiting step present in regular forward translocation may not exist, and the entire translocation process may be consistent with the Brownian ratchet mechanism as the stability of the intermediate state is likely comparably to that of the pre- and post-translocation states. However, altering the free energy landscape is probably not beneficiary to the overall efficiency of the NAC. The about 50-fold increase in *K*_M,NTP_ likely indicates the impairment of the precise placement of the +1 nucleotide by the mutant RdRP. Based on these analyses, we propose that other processive nucleic acid polymerases may also need to stringently control the translocation to bias the pre- and post-translocation state for efficient NTP binding and catalysis, through certain interactions to ensure the positional control of the templating nucleotide (i.e., the +1 template nucleotide). While the capture of three types of translocation intermediates in the EV71 RdRP allowed us to reveal the motif G hurdle residues as the key component in the stringent control, solving analogous intermediate structures may be needed to reveal functionally equivalent polymerase component(s) in other classes of nucleic acids polymerases systems. In bacterial DNA-dependent RNA polymerase, a pausing-related structure shows a conformation similar to the S_6B_ in this study and our previously reported S_6_ structure, with an overall pre-translocated template DNA and post-translocated product RNA^20^. The retention of the template strand in this structure is likely related to the interactions between the nucleotides at the +1/+2 junction and the bridge helix, which also plays an essential role in catalysis. Translocation-related structures with an intermediate positioning of the +1 template nucleotide and the downstream DNA were reported in eukaryotic RNA polymerase II (Pol II) and bacterial RNA polymerase with a kinked bridge helix partially occupying the +1 site^21,22^. However, intermediate structure with a kinked bridge helix and a translocating template-product duplex, analogous to the S_6M_ structure in this study, have not been observed in either systems.

Motif G has been only present in viral RdRPs, and even the most homologous reverse transcriptase does not have such an element. Our data here demonstrate that the motif G hurdle residues are tunable to some extent with small-side-chain residue replacement. Therefore, these two residues may serve as RdRP-unique variable sites to alter the RdRP properties as well as the virus behavior, and could provide opportunity for potential applications related to RNA virus reverse genetics and vaccine development. We previously suggested that motifs A/D movement essential for catalysis is not coupled to translocation^23^. However, the observation of catalytic efficiency modulation by mutation(s) in the hurdle residues suggests that motif G not only serves as the key element to control translocation but also have indirect impact on catalysis. Additionally, The motif F isoleucine (EV71 RdRP I176, with leucine or alanine in RdRPs from a minority of RNA viruses), that caps the +1 template base in the pre- and post-translocation states, moved with the base during template translocation as suggested by the S_6M_ structure (Fig. 5c). The motif B loop, which contains an absolutely conserved glycine (G290 in EV71 RdRP) and was proposed in PV and influenza virus studies as a candidate to “push” the nascent base pair toward upstream during translocation^24–26^, undergoes small-scale backbone shift (no greater than 1 Å) in accordance with motifs G and F (Fig. 5d, and Supplementary Fig. 5 and Movie 1). This loop may indeed fully swing toward the upstream in the final stage of translocation as suggested by the “out”-conformation captured using PV RdRP constructs bearing mutations in this region^24^. Taken together, as some RdRP components contribute to both catalysis and translocation, these two key steps of NAC are coupled to some extent and the modulation of translocation independent of catalysis may not be readily achieved.

While efficient and rigorously controlled catalysis and translocation ensures the long chain nucleic acid biosynthesis in a processive manner, proofreading activities are necessary for maintaining an optimal fidelity level in genome replication, transcription, and reverse transcription processes. Under certain circumstances in transcription (e.g., pausing or arrest), the 3′-end nucleotide can be cleaved through hydrolysis as assisted by factors such as bacterial GreA/GreB and eukaryotic TFIIS^27,28^, or through pyrophosphorolysis reactions^29,30^. These activities, either directly linked to proofreading or related to iterative cleavage and re-synthesis processes beneficiary to the formation of ECs, may require a reverse translocation (known as “backtracking” in transcription) movement of the template-product to position the 3′-end nucleotide in the +1 position for cleavage reactions. The in-crystal reverse translocation and the intermediate structure observed in this study provide a structural basis to understand this movement (Supplementary Fig. 2, Fig. 3, and Supplementary Movie 2). The pyrophosphorolysis is often much slower than nucleotide addition. When comparing the forward and reverse translocation intermediate structures S_6A_/S_6B_ and S_6RA_/S_6RB_, a major difference is the disruption and reestablishment of baseparing interactions during the reverse translocation, suggesting that the reverse translocation is relatively difficult to achieve (compare Supplementary Movies 1 and 2). We performed PP_i_ induced cleavage in solution and found that the cleavage only become obvious when PP_i_ concentration reached 2 or 5 mM when Mg^2+^ or Mn^2+^ was provided as the divalent metal ion, respectively (Supplementary Fig. 7, lanes 11–19 and 34–39). Although multiple cleavage events were evident, very slow kinetics and low yield were observed in the presence of either divalent metal ion (Supplementary Fig. 7b). This is also largely consistent with the crystallographic observation of reverse translocation but not cleavage (Fig. 3 and Supplementary Fig. 2).

RNA virus genome replication and transcription are typically not regulated by proofreading factors, except that the exonuclease activity may be related to fidelity regulation in coronaviruses and mismatched NTP-mediated 3′-end nucleotide cleavages were observed in hepatitis C virus RdRP^31,32^. However, intrinsic cleavage activities could still exist either through hydrolysis and pyrophosphorolysis under certain situation for viral RdRPs to achieve its optimal synthesis and fidelity. We hypothesize that misincorporation or sequence-related pausing may alter the free energy landscape of translocation, in particular for the stability of the post-translocation state, thus increasing the opportunity for reverse translocation and subsequent cleavage. In this way, the polymerase may improve the overall synthesis efficiency and fidelity without the assistance of other protein factors.

## Methods

### Plasmid construction and protein preparation

Two pET26b-Ub vector-based plasmid containing the EV71 (strains SK-EV006-LPS1 and HeN09-17/HeN/CHN2009) *3D*^pol^ (RdRP) gene were used as the original cloning templates to construct the mutant plasmids according to previously described methods^13,33,34^. The constructs derived from the first strain were used for crystallographic studies, while those derived from the second strain were used in biochemical characterizations. Cell growth, isopropyl-β-D-thiogalactopyranoside (IPTG) induction, cell harvesting, cell lysis, protein purification, and protein storage were performed as described previously^13,16^.

### RNA preparation and 3D^pol^ EC assembly

The 35-mer template (T35) RNA used was prepared by T7 RNA polymerase-glmS ribozyme-based approaches described previously^35,36^, and the 10-mer RNA primer (P10) was chemically synthesized (Integrated DNA technologies). RNA construct assembly and EC assembly, purification, and storage were performed as previously described^13^, except that the KCl and NaCl concentrations were 50 and 20 mM, respectively, and MES (pH 6.5) was used as the buffering agent for EC assembly and storage for PDB entries 6LSE and 6LSF, and the NaCl concentration was further adjusted to 30 mM, and HEPES (pH 7.0) was used as the buffering agent for PDB entries 6LSG and 6LSH.

### EC crystallization and EC crystal soaking trials

The EC crystals were grown by sitting-drop vapor diffusion at 16 °C using 10 mg/mL EC samples. Crystals grew to their final size within 1-2 weeks in a precipitant solution containing 0.17 M ammonium sulfate, 0.085 M MES (pH 6.5), 25.5% (wt./vol.) PEG5000 monomethyl ether, and 15% (vol./vol.) glycerol. Crystal soaking trials were done using the precipitant solution supplemented with 5 mM reactant each (NTP(s) or PP_i_) and 10 mM divalent metal ions (MgCl_2_ or MnCl_2_). Crystals were directly cooled and stored in liquid nitrogen prior to data collection. In order to minimize RNA degradation, diethyl pyrocarbonate (DEPC)-treated water was used in the RNA preparation, the EC assembly, purification, and crystallization wherever possible and practical.

### Crystallographic data processing and structure determination

X-ray diffraction data was collected at Shanghai Synchrotron Radiation Facility (SSRF) beamline BL19U1 (wavelength: 0.9789 Å) for the S_6A_/S_6B_ structure and BL17U1 (wavelength: 0.9792 Å) for S_6RA_/S_6RB_ and S_6M_ (C_0_ and C_2_) structures at 100 K. Data of at least 180° were typically collected in 0.2° oscillation steps. Reflections were integrated, merged and scaled using HKL2000 (Table 1)^37^. The initial structure solution was obtained using the molecular replacement program PHASER^38^ with coordinates derived from EV71 EC structures (PDB entries 5F8G/5F8L, chains A-C) as the search model^13^. Manual model building and structure refinement were done using Coot and Phenix, respectively^39,40^. For the S_6A_/S_6B_ and S_6RA_/S_6RB_ structures, the entire product RNA chain was split into two alternate conformations and group occupancy refinement was applied. Ramachandran statistics are 92.7/7.1/0.0/0.2, 92.4/7.3/0.0/0.2, 94.4/5.4/0.0/0.2, and 93.2/6.6/0.0/0.2 for the S_6A_/S_6B_, S_6RA_/S_6RB_, S_6M_ (C_0_), and S_6M_ (C_2_) structures, respectively (values are in percentage and are for most favored, additionally allowed, generously allowed, and disallowed regions in Ramachandran plots, respectively). The 3500-K composite simulated-annealing (SA) omit 2F_o_-F_c_ electron density maps were generated using CNS^41^. Unless otherwise indicated, protein structure superimposition was done using the maximum likelihood-based structure superpositioning program THESEUS^42^.

### The stopped-flow fluorescence-based single nucleotide incorporation assay

This assay was modified from protocols described previously^16,43^. Briefly, the 10-mer RNA primer (P10) and the 33-mer RNA template T33-F_int_ with an internal fluorescein label (Integrated DNA technologies) were annealed at a 1.1:1 molar ratio to yield the T33-F_int_/P10 construct that was used for EC assembly and stopped-flow trials, except that: the enzyme concentration was 16 μM for EC assembly; the NaCl, KCl, MgCl_2_, and TCEP concentrations in the SF buffer were adjusted to 30, 50, 5, and 5 mM, respectively; the fluorescence excitation monochromator bandwidth is 4 nm. The fluorescence signal change was measured at 22.5 °C after rapid-mixing, producing a final solution with 25 nM RNA, 400 nM 3D^pol^, 0.5 μM ATP/GTP and different concentrations of CTP in SF buffer. The EC was assembled using the T33-F_int_/P10 and EV71 RdRP upon incorporation of a G-A-G-A tetra-nucleotide with GTP and ATP as only NTP substrates, producing a 14-mer (P14)-containing EC (EC14). A rapid fluorescence signal decrease was observed when CTP was mixed with EC14 using a stopped-flow instrument (Applied PhotoPhysics Chirascan SF3).

Data analysis and fitting were done as described previously^16^. Briefly, the data of rapid signal decrease observed for CMP incorporation were fitted into a single exponential decay curve Y = amplitude•exp(-rate•X) + offset. The incorporation rates were then plotted as a function of the CTP concentration and the data were fitted to the Michaelis-Menten type equation, rate=*k*_cat_•[CTP]/([CTP] + *K*_M,CTP_), to determine the *k*_cat_ and apparent *K*_M,CTP_ values for calculating the catalytic efficiency (*k*_cat_/*K*_M,CTP_).

### Reporting summary

Further information on research design is available in the Nature Research Reporting Summary linked to this article.

## Supplementary information

Supplementary Information

Peer Review File

Reporting Summary

Description of Additional Supplementary Files

Supplementary Movie 1

Supplementary Movie 2

## Data Availability

Atomic coordinates and structure factors for the reported crystal structures have been deposited with the Protein Data bank under accession numbers PDB 6LSE [10.2210/pdb6LSE/pdb], PDB 6LSF [10.2210/pdb6LSF/pdb], PDB 6LSG [10.2210/pdb6LSG/pdb], and PDB 6LSH [10.2210/pdb6LSH/pdb]. The authors declare that the data that support the findings of this study are available within this paper and its supplementary information files, and are available from the corresponding author on reasonable request.

## Source data

Source Data
