# Supplementary material for: Stringent control of the RNA-dependent RNA polymerase translocation revealed by multiple intermediate structures
Source: Nat Commun. 2020 May 25;11:2605. doi: 10.1038/s41467-020-16234-4 (PMC7248106; doi:10.1038/s41467-020-16234-4)
Supplement: Supplementary file 1 — Supplementary Information [file 41467_2020_16234_MOESM1_ESM.pdf]

Supplementary information

Stringent control of the RNA-dependent RNA polymerase translocation revealed by multiple intermediate structures

Meihua Wang, Rui Li, Bo Shu, Xuping Jing, et al.

This Supplementary Information includes:

Supplementary Figures 1-7

Supplementary Table 1

Supplementary Figures and Figure Legends

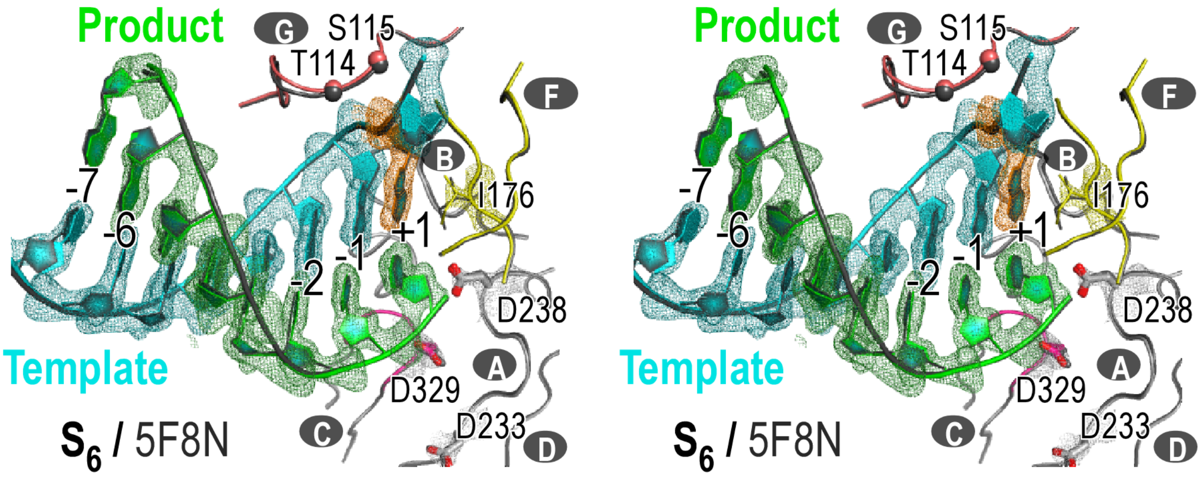

Supplementary Figure 1. An EV71 RdRP C291M mutant-derived translocation intermediate structure exhibited nearly identical conformation to a previously reported state 6 structure. Stereo-pair images of the superposition of a C291M-derived S_6 intermediate structure (color) and the previously reported WT RdRP-derived translocation intermediate structure (dark grey, PDB entry 5F8N) [1]. A C291M-derived $2F_o$-F_c electron density map (contoured at 2.0 σ) is overlaid with the corresponding model. Coloring scheme: template in cyan (+1 nucleotide in orange), product in green, palm in grey (YGDD sequence in magenta), ring finger subdomain in yellow, motif G in pink. Capital letters with grey background indicate corresponding RdRP motifs. The α-carbon atoms of the two critical motif G residues, T114 and S115, are shown as spheres. The side chains of conserved residues I176 in motif F, D233 and D238 in motif A, and D329 in motif C are shown as sticks.

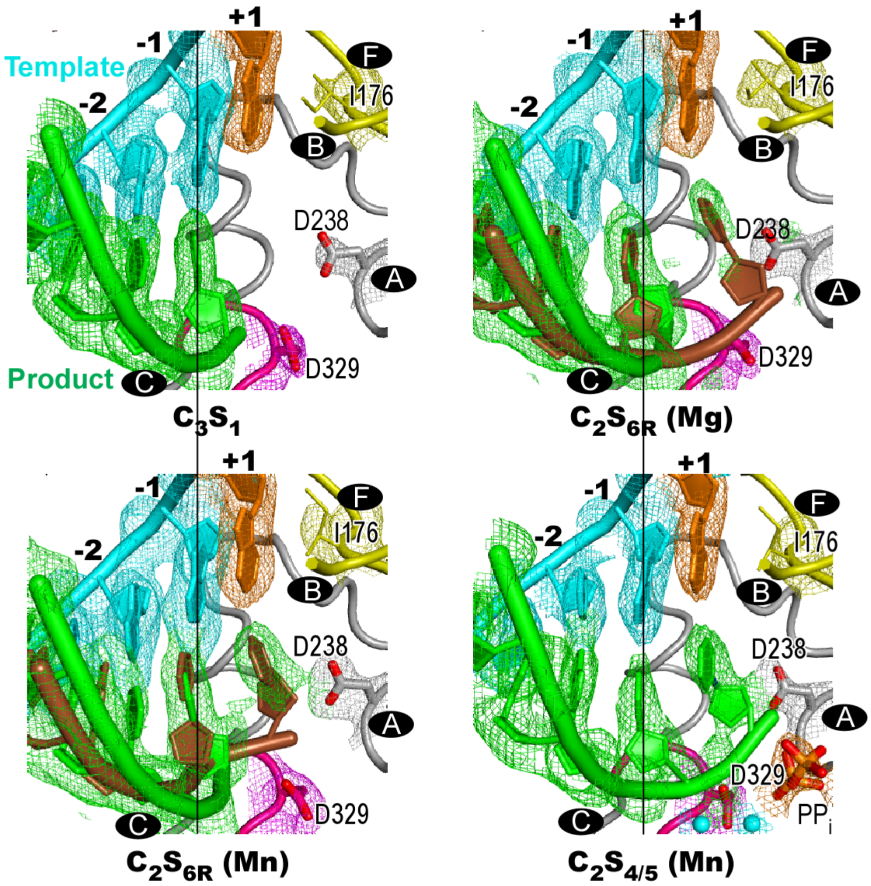

Supplementary Figure 2. Time-resolved crystallography illustrating RdRP reverse translocation. Top left: A post-translocation state structure (C_3S_1) obtained after RdRP EC soaking trials in the presence of CTP. Bottom left: A C_3S_1-derived reverse translocation intermediate structure (C_2S_{6R} (Mn)) upon 2-min soaking with pyrophosphate (PP_i) and $MnCl_2$. Top right: A C_3S_1-derived reverse translocation intermediate structure (C_2S_{6R} (Mg)) upon 20-min soaking with PP_i and $MgCl_2$. Bottom right: A C_3S_1-derived pre-translocation state structure ($C_2S_{4/5}$) upon 10-min soaking with PP_i and $MnCl_2$. The assignment of the partially closed pre-translocation state $S_{4/5}$ was based on the comparison of this structure with reference state 4 (closed active site, PDB entry 5F8J) and reference state 5 (open active site, PDB entry 3OL9, chain A complex) structures (Fig. 1b) [1,2]. The manganese ions are shown as cyan spheres. The structural models were overlaid with $2F_o$-F_c electron density maps contoured at 1.5 (pre- and post-translocation states) or 0.8 (intermediates) σ. The coloring scheme is the same as in Fig. 2b.

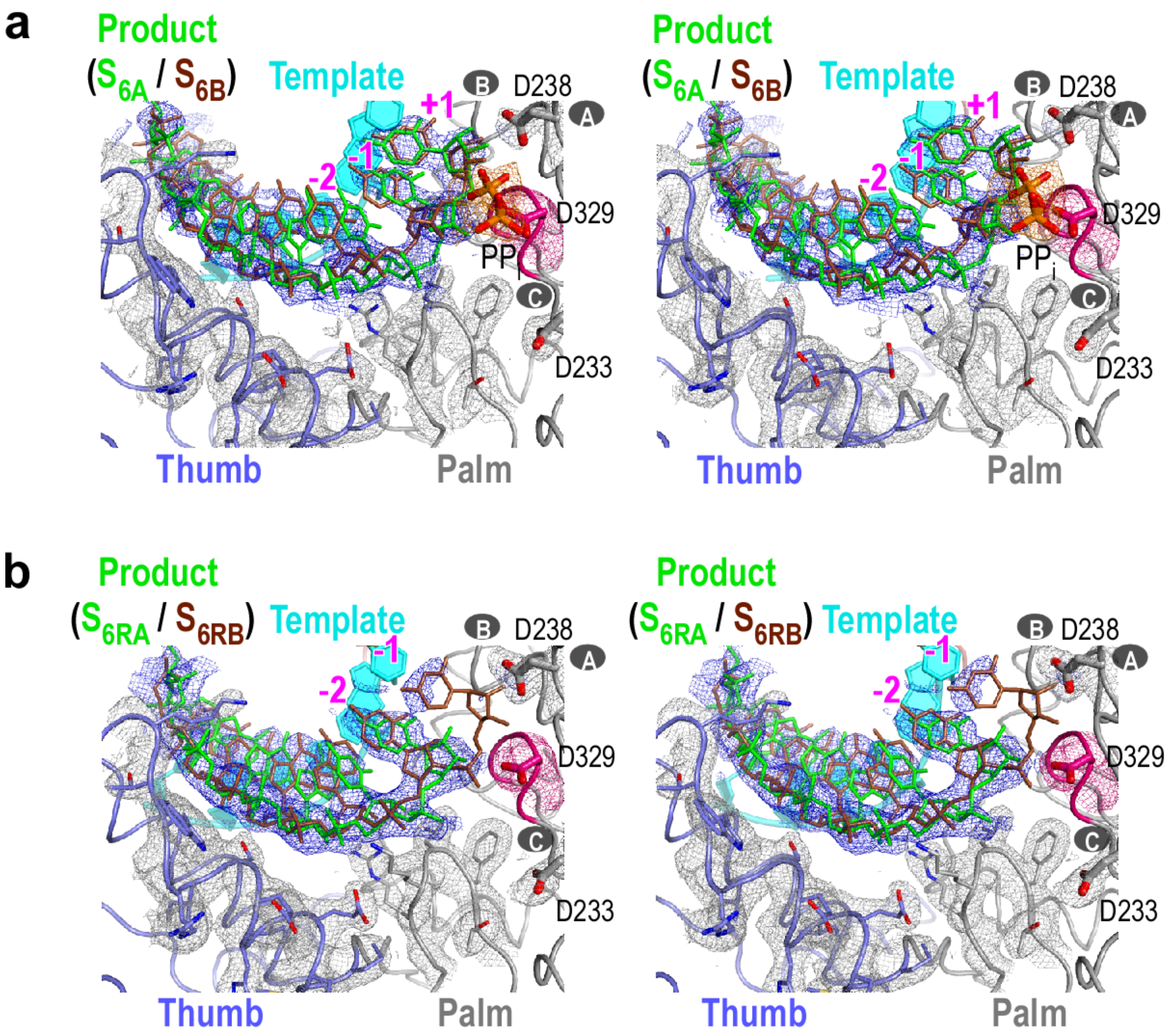

Supplementary Figure 3. The heterogeneity of the product strand conformation observed in translocation intermediate structure is relatively independent. A-B) Stereo-pair images of the intermediate structure S_{6A}/S_{6B} (A) and S_{6RA}/S_{6RB} (B) with a composite SA omit electron density map (contoured at 0.7 σ for S_{6A}/S_{6B} and 0.8 σ for S_{6RA}/S_{6RB}) overlaid. The coloring scheme and labelling are the same as Fig. 2b and Fig. 3b, except that the nucleotide position was labelled according to the product strand.

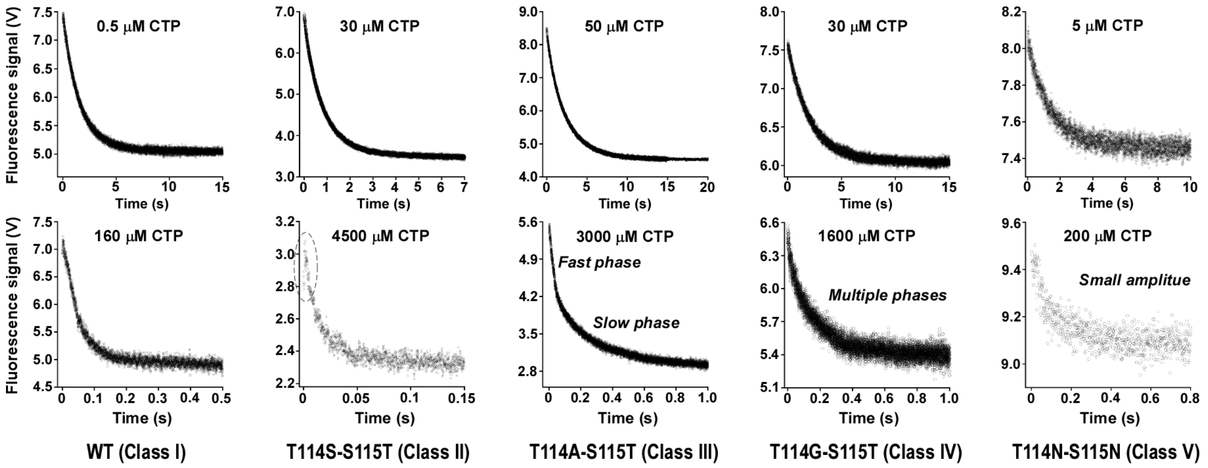

Supplementary Figure 4. Representative kinetics behavior of the five classes of EV71 RdRP constructs. Kinetic traces under low (top) and high (bottom) concentrations of CTP for five RdRP constructs representing different classes of kinetics behaviors in particular under high CTP concentrations. Class I: single exponential decay; Class II: obvious increase (indicated by the dashed oval) followed by single exponential decay; Class III: bi-phasic decay; Class IV: multi-phasic decay; Class V: small amplitude of signal change that may indicate poor yield of EC assembly prior to the stopped-flow trials.

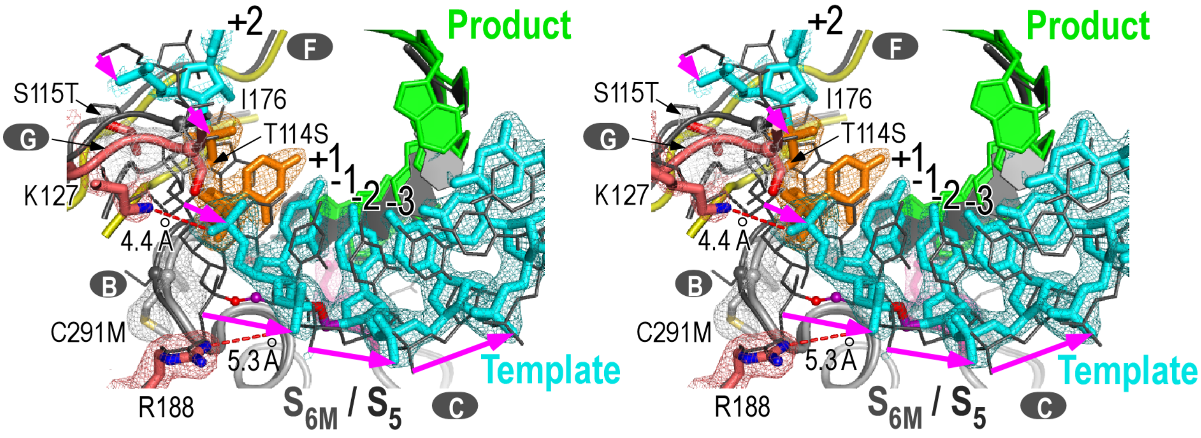

Supplementary Figure 5. Local protein conformational changes in response to global RNA movement during translocation. Stereo-pair images of superposed intermediate structure S_{6M} (color) and reference structure S_5 (dark grey) with the composite SA omit electron density map of the S_{6M} structure (contoured at 1.2 σ) overlaid. The coloring scheme and labelling are the same as Fig. 5d. The 5′- oxygen and carbon atoms of the -2 template nucleotide were shown as red and purple spheres in both structure, highlighting the P-O5′-C5′-C4′ backbone conformation switching from an unusual "S" shape (if compared with those of the neighboring nucleotides) in the S_5 structure to a "Z" shape in the S_{6M} structure due to the upward movement of the -1 template nucleotide. K127 and R188 that form salt bridges (dark grey dashed lines) in the backbone phosphates in the S_5 structure lose such interactions in the S_{6M} structure due to the template movement toward the upstream. The motif B loop moves in accordance with motifs F and G, albeit with smaller distance of the backbone movement (0.8 Å vs. 1.2 Å/1.5 Å for α-carbon distances of motif B residue 291 and motifs F/G residues 176/114 between the superposed structures). The α-carbons of these representative residues are shown as spheres to aid the visualization of coordinated movement of motifs B, F, and G. To aid the visualization of template strand movement, pink arrows are used to indicate the phosphate movement from S_5 to the S_{6M}.

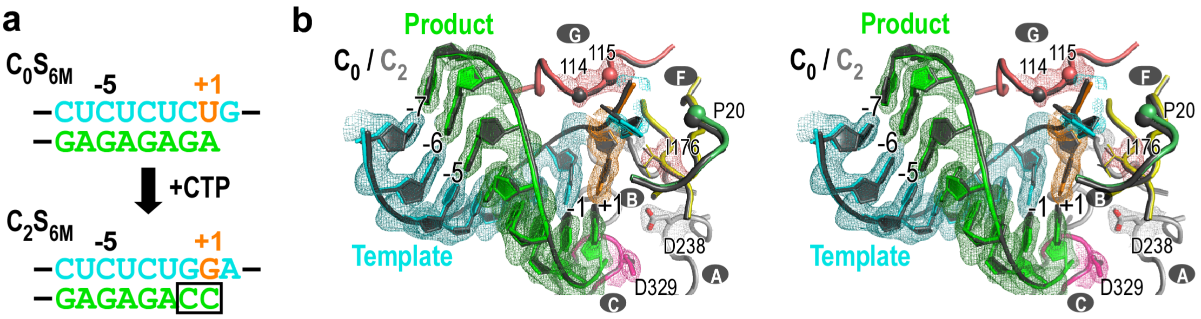

Supplementary Figure 6. The EV71 RdRP ST mutant tended to adopt an intermediate conformation in the late stage of translocation. A) Upon CTP addition in the EC crystal soaking trials, the EC incorporated two CMPs (boxed nucleotides). The template sequence is in cyan with +1 nucleotide in orange. The product sequence is in green. B) Stereo-pair images of the superposed C_0S_6 (dark grey) and C_2S_6 (color) structures with a composite SA omit electron density map (contoured at 1.2 σ) of the C_2S_6 structure overlaid. Compared to the native EC (C_0S_{6M}), the CTP-derived EC (C_2S_{6M}) adopted almost identical conformation. The coloring scheme is the same as in Fig. 5a.

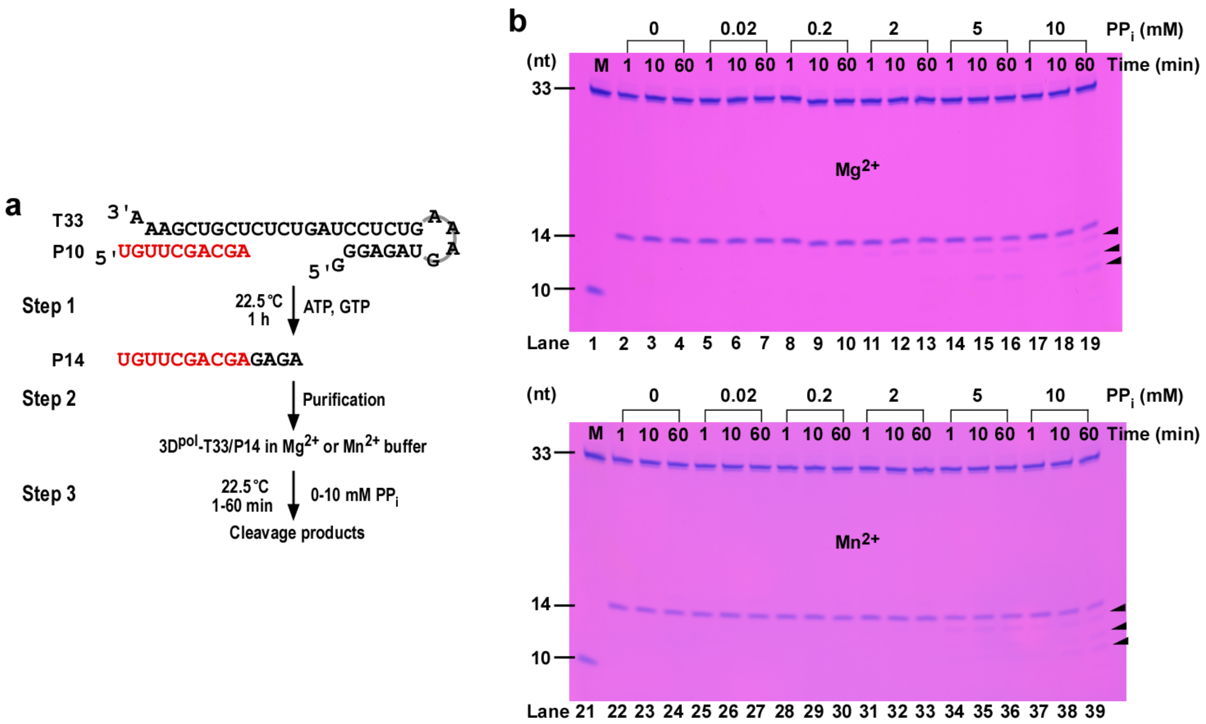

Supplementary Figure 7. PP$_i$-mediated product cleavages in the EV71 RdRP EC. A) The reaction flow chart of the PP$_i$-mediated EC cleavage assays. The EC was assembled using a T33/P10 construct [3]. This construct has otherwise identical sequences to the T33-F$_{int}$/P10 construct used in the stopped-flow assay, except for not having a fluorescein label. Upon addition of GTP and ATP, a 14-mer (P14) containing EC (EC14) was formed. The EC14 was purified using a desalting column to remove free NTPs and was then subjected to treatment with PP$_i$ and divalent metal ions (Mg^{2+} or Mn^{2+}) with a final EC concentration of 4 μM. B) The cleavage reaction mixture was resolved using 20% polyacrylamide gel electrophoresis and the RNA was visualized by Stains-All (Sigma-Aldrich) staining. The T33/P10 construct was used as a marker ("M", lanes 1 and 21). The migration positions of cleavage products were indicated by the solid triangles.

Supplementary references

1. Shu, B. & Gong, P. Structural basis of viral RNA-dependent RNA polymerase catalysis and translocation. *Proc Natl Acad Sci U S A* **113**, E4005-14 (2016).
2. Gong, P. & Peersen, O.B. Structural basis for active site closure by the poliovirus RNA-dependent RNA polymerase. *Proc Natl Acad Sci U S A* **107**, 22505-10 (2010).
3. Wu, J., Lu, G., Zhang, B. & Gong, P. Perturbation in the conserved methyltransferase-polymerase interface of flavivirus NS5 differentially affects polymerase initiation and elongation. *J Virol* **89**, 249-61 (2015).

Supplementary Table

Table S1. Catalytic efficiency analyses of the WT EV71 RdRP and its motif G mutants based on data obtained in a stopped-flow fluorescence single nucleotide incorporation assay.

	Construct	k_{cat} (nt/s)	K_M (μM)	$k_{cat}/K_{M,CTP}$ ($\mu M^{-1} \bullet S^{-1}$)
Class I	WT	22.0±0.2[a]	14.7±0.5[a]	1.50±0.05[b]
	S115G	30.6±0.6	19.5±1.9	1.57±0.16
	S115A	21.6±0.8	14.9±1.9	1.45±0.19
	T114S	33.7±0.6	32.8±1.6	1.03±0.05
	T114A	35.5±0.7	51.2±3.4	0.69±0.05
	T114A-S115A	37.0±0.6	105±5	0.35±0.02
Class II	T114S-S115A	44.7±1.1	57.0±4.4	0.78±0.06
	T114S-S115G	306±28	773±177	0.40±0.10
	T114A-S115G	351±55	2014±610	0.17±0.06
	T114S-S115T	28.8±0.9	765±69	0.04±0.01[d]
Class III	S115T	36.3±1.2	149±13	0.24±0.02
	T114G-S115G	105±25	2760±939	0.04±0.02
	T114A-S115T	8.6±0.2	772±41	0.01±0.01[d]
Class IV	T114G	18.8±0.7	153±21	0.12±0.02
	T114G-S115A	12.9±0.6	463±57	0.03±0.01[d]
	T114G-S115T	8.7±0.6	400±82	0.02±0.01[d]
Class V	T114N-S115N	12.5±0.5	63.9±8.5	0.20±0.03
	T114Y-S115Y	8.5±1.2	351±115	0.02±0.01[d]
Unclassified[c]	T114F-S115F			
	T114L-S115L			

[a] Fitting errors from the Michaelis-Menten curve fitting were provided.

[b] Errors of catalytic efficiency were propagated from the fitting errors of k_{cat} and $K_{M,CTP}$.

[c] The double F and double L mutants had very low yield of EC assembly and therefore were not subjected to stopped flow trials.

[d] The value of 0.01 was used for the propagated fitting error values that are lower than 0.01.
