# Supplementary material for: Stringent control of the RNA-dependent RNA polymerase translocation revealed by multiple intermediate structures
Source: Nat Commun. 2020 May 25;11:2605. doi: 10.1038/s41467-020-16234-4 (PMC7248106; doi:10.1038/s41467-020-16234-4)
Supplement: Supplementary file 3 — Reporting Summary [file 41467_2020_16234_MOESM3_ESM.pdf]

nature research

Corresponding author(s): Peng Gong

Last updated by author(s): Mar 4, 2020

Reporting Summary

Nature Research wishes to improve the reproducibility of the work that we publish. This form provides structure for consistency and transparency in reporting. For further information on Nature Research policies, see Authors & Referees and the Editorial Policy Checklist.

Statistics

For all statistical analyses, confirm that the following items are present in the figure legend, table legend, main text, or Methods section.

n/a	Confirmed	
☒	☐	The exact sample size (*n*) for each experimental group/condition, given as a discrete number and unit of measurement
☒	☐	A statement on whether measurements were taken from distinct samples or whether the same sample was measured repeatedly
☒	☐	The statistical test(s) used AND whether they are one- or two-sided<br>*Only common tests should be described solely by name; describe more complex techniques in the Methods section.*
☒	☐	A description of all covariates tested
☒	☐	A description of any assumptions or corrections, such as tests of normality and adjustment for multiple comparisons
☒	☐	A full description of the statistical parameters including central tendency (e.g. means) or other basic estimates (e.g. regression coefficient) AND variation (e.g. standard deviation) or associated estimates of uncertainty (e.g. confidence intervals)
☒	☐	For null hypothesis testing, the test statistic (e.g. *F*, *t*, *r*) with confidence intervals, effect sizes, degrees of freedom and *P* value noted<br>*Give P values as exact values whenever suitable.*
☒	☐	For Bayesian analysis, information on the choice of priors and Markov chain Monte Carlo settings
☒	☐	For hierarchical and complex designs, identification of the appropriate level for tests and full reporting of outcomes
☒	☐	Estimates of effect sizes (e.g. Cohen's *d*, Pearson's *r*), indicating how they were calculated

Our web collection on statistics for biologists contains articles on many of the points above.

Software and code

Policy information about availability of computer code

Data collection	Single crystal X-ray diffraction data collection was performed using Blu-Ice 5.0 software. The stopped-flow fluorescence data were collected using the ProData ChiraScan software (Applied Photophysics).
Data analysis	X-ray diffraction data processing was performed using HKL2000 v716.1 software. CCP4 6.5, PHENIX 1.10, and CNS 1.3_r8 were used for model building, refinement, map generation, and validation. Maximum likelihood structure superimposition was done using Theseus 2.0.1. Curve fitting of the stopped-flow fluorescence data was performed using KaleidaGraph 4.1.3.

For manuscripts utilizing custom algorithms or software that are central to the research but not yet described in published literature, software must be made available to editors/reviewers. We strongly encourage code deposition in a community repository (e.g. GitHub). See the Nature Research guidelines for submitting code & software for further information.

Data

Policy information about availability of data

All manuscripts must include a data availability statement. This statement should provide the following information, where applicable:

- Accession codes, unique identifiers, or web links for publicly available datasets
- A list of figures that have associated raw data
- A description of any restrictions on data availability

The atomic coordinates and structure factors for the reported viral RNA-dependent RNA polymerase translocation intermediate crystal structures have been deposited in the Protein Data Bank under accession numbers 6LSE, 6LSF, 6LSG, and 6LSH. The authors declare that key biochemical data that support the findings of this study are available within this paper and its supplementary information files, and additional data are available from the corresponding author on reasonable request.

Field-specific reporting

Please select the one below that is the best fit for your research. If you are not sure, read the appropriate sections before making your selection.

☒ Life sciences ☐ Behavioural & social sciences ☐ Ecological, evolutionary & environmental sciences

For a reference copy of the document with all sections, see nature.com/documents/nr-reporting-summary-flat.pdf

Life sciences study design

All studies must disclose on these points even when the disclosure is negative.

Sample size	n/a
Data exclusions	No data was excluded.
Replication	The X-ray diffraction data collection was designed to ensure the high enough redundancy, and all diffraction datasets reported in this study have a redundancy value higher than 4.0. For the stopped-flow experiments, every kinetic trace reported in this study is an average of at least five measurements.
Randomization	n/a
Blinding	n/a

Reporting for specific materials, systems and methods

We require information from authors about some types of materials, experimental systems and methods used in many studies. Here, indicate whether each material, system or method listed is relevant to your study. If you are not sure if a list item applies to your research, read the appropriate section before selecting a response.

Materials & experimental systems

n/a	Involved in the study	
☒	☐	Antibodies
☒	☐	Eukaryotic cell lines
☒	☐	Palaeontology
☒	☐	Animals and other organisms
☒	☐	Human research participants
☒	☐	Clinical data

Methods

n/a	Involved in the study	
☒	☐	ChIP-seq
☒	☐	Flow cytometry
☒	☐	MRI-based neuroimaging
