# Supplementary material for: Stringent control of the RNA-dependent RNA polymerase translocation revealed by multiple intermediate structures
Source: Nat Commun. 2020 May 25;11:2605. doi: 10.1038/s41467-020-16234-4 (PMC7248106; doi:10.1038/s41467-020-16234-4)
Supplement: Supplementary file 4 — Description of Additional Supplementary Files [file 41467_2020_16234_MOESM4_ESM.docx]

**Description of Additional Supplementary Files**

File name: Supplementary Movie 1
Description: A modelled conformational transition in forward translocation. The video starts with an EV71 RdRP EC pre-translocation structure (S_4/5,_ PDB entry 5F8M), through the first intermediate structure (S6, PDB entry 5F8N) and then the second intermediate structure (S_6M_, 6LSH), and finishes with a post-translocation structure (S_1_, 5F8L). The duration of each of these three segments is equal. The RdRP chains in the structures were superposed using THESEUS ^1^. The coloring scheme is the same as in Fig. 5a with thumb and middle finger subdomains in slate and orange, respectively. The α-carbon atoms of residues P20 (green), 114 (T or S, in pink), 115 (S or T, in pink), I176 (in yellow), 291 (C or M, in gray), and the phosphorus atoms of both RNA strands (template in cyan with +1 in orange and product in green) were shown as spheres to aid the visualization of the RNA movement relative to the polymerase and notable polymerase conformational changes during translocation. In order to generate this video, the nucleotides of the last two states (6LSH and 5F8L) were mutated to have them identical to those in the first two states (5F8M and 5F8N). The video was generated using PyMOL (The PyMOL Molecular Graphics System, Schrödinger, LLC).

File name: Supplementary Movie 2
Description: A modeled conformational transition in reverse translocation. The video starts with an EV71 RdRP EC post-translocation structure (S_1_, PDB entry 5F8L) and finishes with a reverse translocation intermediate post-translocation structure (S_6RB,_ 6LSF). The RdRP chains in the structures were superposed using THESEUS. The coloring scheme and the usage of the sphere representations are the same as in Supplementary Movie 1. The video was generated using PyMOL.

**References** 1. Theobald, D.L. & Wuttke, D.S. THESEUS: maximum likelihood superpositioning and analysis of macromolecular structures. *Bioinformatics* **22**, 2171-2 (2006).
