# Supplementary material for: Stringent control of the RNA-dependent RNA polymerase translocation revealed by multiple intermediate structures
Source: Nat Commun. 2020 May 25;11:2605. doi: 10.1038/s41467-020-16234-4 (PMC7248106; doi:10.1038/s41467-020-16234-4)
Supplement: Supplementary file 2 — Peer Review File [file 41467_2020_16234_MOESM2_ESM.pdf]

Reviewers' comments:

Reviewer #1 (Remarks to the Author):

Synthesis of nucleic acids is a critical process for all life forms and is performed by nucleic acid polymerases that processively catalyse multiple rounds of template directed nucleotide addition. This manuscript reveals important new details of the nucleotide addition cycles (NAC) based on high resolution crystal structures of the enterovirus 71 RNA-dependent RNA polymerase (RdRP), which is active for several NACs in the crystal.

Previous studies from the Gong lab on the same system have described six steps in the NAC, including both open and closed pre-translocation states of the active site, post-translocated states after pyrophosphate release and an intermediate state, where the product is partially translocated. The manuscript by Wang et al takes this work further and describes several X-ray structures that capture how product and especially the template translocation occurs, an aspect that has so far been poorly understood. In particular, using mutants in RdRP conserved motif G, which allow trapping of a state where the template has partially translocated, they show that motif G constitutes a hurdle to be overcome during translocation. They suggest that motif G's role is to prevent sloppiness in template positioning and thus promote efficient catalysis. In addition, they reveal an intermediate in the pyrophosphate induced reverse translocation of the product and discuss the implications for proofreading.

The authors have maximally exploited the versatility of their system to capture new translocation states and add insightful details to their previous observations. Moreover, the mechanisms proposed probably apply generally to other viral RdRPs and may aid antiviral drug development. Thus the manuscript is worthy of publication. While the analysis is generally well argued, some aspects require clarification.

1. The authors describe several structures with alternative conformations of the nucleic acid strands visible. However, they do not comment whether strand movement is accompanied also by protein movement. Are alternative positions of protein residues seen and modelled? Are the residues interacting with the product static, or do they follow the product strand, in other words, is the product floating in a static environment or does the protein 'paddle' the product strand?

2. The authors should discuss whether any other parts of the active site might facilitate the product strand movement. For example, in influenza polymerase the conserved Met rich motif B loop, has been proposed to push on the product strand, in a 'stroke' like fashion (Kouba et a NSMB, 2019). Is it possible, that the equivalent of motif B in enterovirus 71 could play a similar role? Do authors observe any significant and perhaps coordinated movement of motif F (in particular Ile176) together with motif B in the translocation structures? How does the Ile176 move out of the way to allow the +2 base to move into the +1 position and then back to support the +1 base? A more detailed figure of the active site motifs movement should be included to accompany Figure 4A and 4C and perhaps the discussion on lines 310-313 extended.

3. The authors describe motif G Thr114 and Ser115 as the 'hurdle' for the template translocation, but how is the hurdle overcome? A relatively conserved positively charge residue just preceding these two residues (His113 in EV71, positively charged residue -ve strand RNA viruses) directly interacts with product strand at position -6 Is it possible that His113 senses product translocation and induces some movement of the G motif Thr114 and Ser115 that releases of the 'hurdle' and trigger template translocation? If so, might it be interesting to enzymology characterize His113 mutants?

4. Lys127/Arg188, contacting the template strand in the pre-translocation state, are packed over the G-motif to reach the template. That means that when motif G moves, Lys127/Arg188 might be shifted away, loosening the contact with template, thus releasing it to translocate. Could authors comment on

such a scenario?

5. After the ‘chemistry’ step, the presented results show the product strand translocases first, thus leaving the +1 incoming NTP position empty. Is it possible that the incoming NTP insertion towards the partially moved -1 template position, and subsequent base-paring in +1 position might play a role in completing template translocation?

Minor comments:
Line 56-57. Could authors compare the ‘push’ mechanism with recent findings about motif B in transcriptionally active influenza B strain RdRP (Kouba et a NSMB, 2019).
Line 14-16: Not very clear.
Line 57: Change to: An analogous
Line 90. Explain results from the ‘tested’ C291M mutation or reword.
Lines 123-125: Relative high level of freedom of the template contradicts line 107-108 from previous paragraph.
Line 391: Explain the principle of the assay in one sentence.
Figure S4. The difference between the experiments to obtain C0S6M and C2S6M is not very clear. The template RNAs should be listed in some table and incorporated nucleotides in the reaction should be marked.

Reviewer #2 (Remarks to the Author):

Summary
The manuscript by Wang, et al. characterizes translocation intermediates for viral RNA-dependent RNA polymerase (RdRP) elongation complexes (EC) during the nucleotide addition cycle (NAC). This study provides insight into the forward and reverse translocation events, the role of RdRP motif G, and a transition state during translocation. The study uses X-ray crystallography to solve structures of enterovirus 71 (EV71) RdRP ECs and stopped-flow fluorescence-based single nucleotide incorporation assay to explore the role of RdRP motif G in controlling template RNA strand movement. The combination of structures with enzymology of mutant RdRP provides a convincing model of translocation and identify the residues that are important in this step. The data are compelling and appear rigorous. The findings are also significant as the molecular details of translocation by RNA and DNA dependent RNAPs are still fairly uncharacterized. I do have some critiques, and they are listed below. I recommend publishing upon revision.

The aims of this study are listed below and were demonstrated convincingly:
1. Structural elucidation of EV71 RdRP ECs translocation states
2. Examination of the role of the RdRP motif G in translocation

The approaches used to address these aims include:

1. X-ray crystallography to determine the structures of EV71 RdRP ECs using NTP incorporation and pyrophosphate (PPi) to capture forward and reverse translocation states
2. Characterization of the motif G hurdle residues using mutational analysis and stopped-flow fluorescence-based single nucleotide incorporation assays
3. X-ray crystallography to determine the structural consequences of a mutation in the motif G for template strand restriction during translocation

Major critiques

1. In the introduction, the authors claim there is no structural evidence of translocation intermediates with a kinked BH. That is not true. Brueckner & Cramer (NSMB, 2008) published a paper describing a structure of a possible pol II translocation intermediate. Weixlbaumer (Cell 2013) also determined a structure with relevant similarities to the Brueckner/Cramer structure. This does not take away from the significance of this work but should be mentioned and this work should be discussed in the context of those studies in the intro and discussion.

2. I think the NAC needs to be the first figure.

3. Please include a pipeline of the initial template, initial RNA and which NTPs were added to accompany the structures.

4. Because the crystals are a combination of nucleic acid translocation intermediates, the rest of the protein (not interacting with the nucleic acid) should have uniform high resolution around it, can the authors supply some supplemental figures illustrating the rest of the protein represents the high-resolution data.

5. Fig 1B has letters A,C, B and F but are not described in the legend.

6. Authors need to give more background on why they focused T114 and S115 in the results as the hurdle residues. They just delve into "these two positions" (line 174) without much context.

7. Fig. 3 needs to be explained better. The authors assume everyone is familiar with their system. For example, is it just CTP being added? It isn't but is presented such. Does F indicate the fluorophore? Please add a pipeline or include more details in Fig. or legend.

8. Authors do not provide illustrations of the electron density for the motif G hurdle residues (T114-S115) and the basic residues K127/R188 which make key interactions with the nucleic acids. The hurdle resides (T114-S115) are only shown as atomic spheres in already built models (e.g. Figures 4A, 4D, S1B, S4B). The density of the electrostatic interactions between the K127/R188 side chains and the -1/-2 template phosphates is not shown for any of the intermediates. However, authors show electron density for other residues such as I176, D238, and D329 but do not mention them in the main text. The authors should provide electron density figures for residues T114-S115 and K127/R188 to show how they are modeled and how they are interacting with the RNA.

9. The authors outlined a procedure for their RdRP EC crystallization, which involves prolonged incubation of the RNA scaffold in the presence of EV71 RdRP (Main Text, Page 17, Line 368-375). RNAs are susceptible to degradation especially if the protein component of the complex is contaminated with RNases that are carried over during purification. Degradation of the RNA scaffold would have effects on the observed structural intermediates. What measures did the authors use to minimize RNA degradation (e.g RNase-free purification of EV71 RdRP)? Did should run a gel on mature EC crystals to see if the RNA scaffold and product RNA are still intact after crystallization.

10. The main structural focus of the paper is the nucleic acids near the site of catalysis. RNA-RNA (template-product) and nucleic acid/protein interactions with neighboring RdRP motifs are compared and highlighted between the translocation intermediates. However, the authors later state that 'the goal of translocation is to "move" the entire polymerase' (Main Text, Page 11, Line 241) and that the 'conformational changes in translocation are not expected to be localized around the active site' (Main Text, Page 12, Line 243-244). Could the authors elaborate on any conformational changes that they observe in the overall structure of the EV71 RdRP translocation intermediates? Perhaps, the authors can align each structure and provide RMSD values for the comparisons (e.g. overall RP comparisons and nucleic acid/motifs comparisons)?

11. In the S6M structure, the authors show a loss of interactions between K127/R188 and the -1/-2

template phosphates that are usually maintained in the forward (S6A and S6B) and reverse (S6RA and S6RB) translocation intermediates. Authors should expand on the conservation and the roles of the K127/R188 residues. Have the authors performed mutational studies on these residues to assess their roles in catalysis or translocation?

12. The authors capture an intermediate EC state (S6M) by using the T114S-S115T mutant. They proposed the stability of this state is comparable to pre- and post-translocated states (Main Text, Page 11, Line 221-223). To test this, they soaked CTP into the crystal to induce forward translocation (C2) and found that they arrived at the same intermediate state as C0 (absence of CTP) (Main Text, Page 11, Line 223-229). The authors should look at reverse translocation with the S6M intermediate by using PPi and divalent metal ions (similar to what they did to obtain S6RA and S6RB). This would provide evidence that the observed intermediate seen with mutant is between the pathway from pre- to post-translocation (Figure 5C). This is not necessary for this paper, just a friendly suggestion.

Minor comments
1. Main Text, Page 2, Line 14. Change 'that similar strategy' to 'that a similar strategy'.

2. Main Text, Page 2, Line 9. Change ‵regular' to forward'

3. Main Text, Page 3, Line 32. Change 'the understands of the' to 'insights into'.

4. Main Text, Page 5, Line 91. Change 'since equivalent' to 'since the equivalent'.

5. Main Text, Page 6, Line 117. Change 'this type of intermediates' to 'these types of intermediates'.

6. Main Text, Page 10-11, Line 216-217. The authors should define what the 'regular conformation' is and how the -1/-2 template phosphates in the intermediate 'switch' to this conformation. In Figure 4D, the -1/-2 phosphate positions are very different between S5 and S6M.

7. Main Text, Page 11, Line 234. Change 'the 10s-100s-ncleotide per second rapid synthesis in a processive manner' to 'rapid synthesis in a processive manner (tens to hundreds of nucleotides per second)'

8. Main Text, Page 11, Line 240. Remove 'as an entity'.

9. Main Text, Page 12, Line 261. Change 'intermediates for easily' to 'intermediates from easily'.

10. Main Text, Page 13, Line 279-280. Change 'rate-limiting step with high energy barrier comparable to that in the regular translocation may not exist' to 'the rate-limiting step present in regular translocation may not exist'.

11. Main Text, Page 25, Figure 3A. Could the authors comment on the conservation of G117 in the motif G? Did they also mutate this residue in their enzymological studies?

12. Supplementary Information, Page 3, Figure S2 (bottom-right). C2S4/5 should be labeled with '(Mn)' since it was generated using PPi and MnCl2.

13. Supplementary Information, Page 4, Figure S3, Class V. 'amplitue' should be corrected to 'amplitude'.

14. Supplementary Information, Page 5, Figure S4. Show electron density for the C2S6M map similar to Figures 1B, 2B, 4A, S2.

15. Supplementary Information, Page 7, Supplementary Video Legends. Provide timestamps for each

structure shown in the video. For clarity, authors should label the states and key residues and bases in Video S1 and Video S2.

Point-by-point responses to reviewers' comments for manuscript tracking #: NCOMMS-20-07789-T

Stringent control of the RNA-dependent RNA polymerase translocation revealed by multiple intermediate structures

by Meihua Wang, Rui Li, Bo Shu, Xuping Jing, Han-Qing Ye, Peng Gong

(Our responses are shown in boldface text with grey background)

Reviewers' comments:

Reviewer #1 (Remarks to the Author):

Synthesis of nucleic acids is a critical process for all life forms and is performed by nucleic acid polymerases that processively catalyse multiple rounds of template directed nucleotide addition. This manuscript reveals important new details of the nucleotide addition cycles (NAC) based on high resolution crystal structures of the enterovirus 71 RNA-dependent RNA polymerase (RdRP), which is active for several NACs in the crystal.

Previous studies from the Gong lab on the same system have described six steps in the NAC, including both open and closed pre-translocation states of the active site, post-translocated states after pyrophosphate release and an intermediate state, where the product is partially translocated. The manuscript by Wang et al takes this work further and describes several X-ray structures that capture how product and especially the template translocation occurs, an aspect that has so far been poorly understood. In particular, using mutants in RdRP conserved motif G, which allow trapping of a state where the template has partially translocated, they show that motif G constitutes a hurdle to be overcome during translocation. They suggest that motif G's role is to prevent sloppiness in template positioning and thus promote efficient catalysis. In addition, they reveal an intermediate in the pyrophosphate induced reverse translocation of the product and discuss the implications for proofreading.

The authors have maximally exploited the versatility of their system to capture new translocation states and add insightful details to their previous observations. Moreover, the mechanisms proposed probably apply generally to other viral RdRPs and may aid antiviral drug development. Thus the manuscript is worthy of publication. While the analysis is generally well argued, some aspects require clarification.

We thank this reviewer for all the comments and concerns and our responses are listed below.

1. The authors describe several structures with alternative conformations of the nucleic acid strands visible. However, they do not comment whether strand movement is accompanied also by protein movement. Are alternative positions of protein residues seen and modelled? Are the residues interacting with the product static, or do they follow the product strand, in other words, is the product floating in a static environment or does the protein 'paddle' the product strand?

In response to this comment and the 4th point of the second reviewer, we have provided the Supplementary Figure 3 to show conformation uniformity of the product-interacting protein regions. We have added descriptions in the results section to comment on this observation.

2. The authors should discuss whether any other parts of the active site might facilitate the product strand movement. For example, in influenza polymerase the conserved Met rich motif B loop, has been proposed to push on the product strand, in a 'stroke' like fashion (Kouba et a NSMB, 2019). Is it possible, that the equivalent of motif B in enterovirus 71 could play a similar role? Do authors observe any significant and perhaps coordinated movement of motif F (in particular Ile176) together with motif B in the translocation structures? How does the Ile176 move out of the way to allow the +2 base to move into the +1 position and then back to support the +1 base? A more detailed figure of the active site motifs movement should be included to accompany Figure 4A and 4C and perhaps the discussion on lines 310-313 extended.

We have used the new Supplementary Fig. 5 and Video 1 to complement the Fig. 5 in the main text. Small-scale backbone movement of motif B in accordance with motifs F/G is discussed in the context of the proposals both in the influenza study (Kouba at al. NSMB 2019) and the previous PV study

(Sholders and Peersen, JMB 2014) is comment and the 4th point of the second reviewer, we have provided the Supplementary Figure 3 to show conformation uniformity of the product-interacting protein regions.

3. The authors describe motif G Thr114 and Ser115 as the ‘hurdle’ for the template translocation, but how is the hurdle overcome? A relatively conserved positively charge residue just preceding these two residues (His113 in EV71, positively charged residue -ve strand RNA viruses) directly interacts with product strand at position -6 Is it possible that His113 senses product translocation and induces some movement of the G motif Thr114 and Ser115 that releases of the ‘hurdle’ and trigger template translocation? If so, might it be interesting to enzymology characterize His113 mutants?

Although we agree that other residues in motif G may also contribute to motif G function, here we want to focus on the 114-115-equivalent sites due to their critical spatial positions for interaction with the template strand. The 113-equivalent site is not conserved in the positive-strand and double-stranded RNA virus RdRPs (Fig. 4A, only 3 basic residues among 14 representative sequences). We would like to explore this and other motif G sites (such as the G117-equivalent site mentioned by reviewer 2) in the future.

4. Lys127/Arg188, contacting the template strand in the pre-translocation state, are packed over the G-motif to reach the template. That means that when motif G moves, Lys127/Arg188 might be shifted away, loosening the contact with template, thus releasing it to translocate. Could authors comment on such a scenario?

The loss of interaction with the backbone phosphate was described in the results section and in Fig. 5D. Here we have added the Supplementary Fig. 5 and highlight this interaction loss by both the interaction distance and the backbone phosphate movement.

5. After the ‘chemistry’ step, the presented results show the product strand translocases first, thus leaving the +1 incoming NTP position empty. Is it possible that the incoming NTP insertion towards the partially moved -1 template position, and subsequent base-paring in +1 position might play a role in completing template

translocation?

We did not observe NTP-like densities in S6M structures. We have mentioned this and commented on this NTP-assisted translocation scenario in the results section.

Minor comments:

Line 56-57. Could authors compare the ‘push’ mechanism with recent findings about motif B in transcriptionally active influenza B strain RdRP (Kouba et a NSMB, 2019).

Please refer to our responses and revision related to the 2nd point.

Line 14-16: Not very clear.

Rephrased.

Line 57: Change to: An analogous

Corrected.

Line 90. Explain results from the ‘tested’ C291M mutation or reword.

Rephrased.

Lines 123-125: Relative high level of freedom of the template contradicts line 107-108 from previous paragraph.

Corrected as "high level of freedom of the product".

Line 391: Explain the principle of the assay in one sentence.

Description has been added in the methods section.

Figure S4. The difference between the experiments to obtain C0S6M and C2S6M is not very clear. The template RNAs should be listed in some table and incorporated nucleotides in the reaction should be marked.

The template RNA sequence has been shown in the new Fig. 1 also in response to point 3 of reviewer 2.Description has been added in the methods section. We have also added a box in panel A to highlight the C-C dinucleotide incorporation that led to the formation of the C2S6M complex.

Reviewer #2 (Remarks to the Author):

Summary

The manuscript by Wang, et al. characterizes translocation intermediates for viral

RNA-dependent RNA polymerase (RdRP) elongation complexes (EC) during the nucleotide addition cycle (NAC). This study provides insight into the forward and reverse translocation events, the role of RdRP motif G, and a transition state during translocation. The study uses X-ray crystallography to solve structures of enterovirus 71 (EV71) RdRP ECs and stopped-flow fluorescence-based single nucleotide incorporation assay to explore the role of RdRP motif G in controlling template RNA strand movement. The combination of structures with enzymology of mutant RdRP provides a convincing model of translocation and identify the residues that are important in this step. The data are compelling and appear rigorous. The findings are also significant as the molecular details of translocation by RNA and DNA dependent RNAPs are still fairly uncharacterized. I do have some critiques, and they are listed below. I recommend publishing upon revision.

The aims of this study are listed below and were demonstrated convincingly:
1. Structural elucidation of EV71 RdRP ECs translocation states
2. Examination of the role of the RdRP motif G in translocation

The approaches used to address these aims include:
1. X-ray crystallography to determine the structures of EV71 RdRP ECs using NTP incorporation and pyrophosphate (PPi) to capture forward and reverse translocation states
2. Characterization of the motif G hurdle residues using mutational analysis and stopped-flow fluorescence-based single nucleotide incorporation assays
3. X-ray crystallography to determine the structural consequences of a mutation in the motif G for template strand restriction during translocation
We thank this reviewer for all the comments and concerns and our responses are listed below.

Major critiques

1. In the introduction, the authors claim there is no structural evidence of translocation intermediates with a kinked BH. That is not true. Brueckner & Cramer (NSMB, 2008) published a paper describing a structure of a possible pol II translocation intermediate.

Weixlbaumer (Cell 2013) also determined a structure with relevant similarities to the Brueckner/Cramer structure. This does not take away from the significance of this work but should be mentioned and this work should be discussed in the context of those studies in the intro and discussion.

We thank this reviewer for pointing out both relevant papers, and we have cited them in the discussion. Our original description, in both introduction and discussion sections, has been modified accordingly.

2. I think the NAC needs to be the first figure.

We have moved the NAC panel of original Fig. S1 to the new Fig. 1 in the main text.

3. Please include a pipeline of the initial template, initial RNA and which NTPs were added to accompany the structures.

We have used panel B in the new Fig. 1 to show the RNA construct and the scheme for EC assembly.

4. Because the crystals are a combination of nucleic acid translocation intermediates, the rest of the protein (not interacting with the nucleic acid) should have uniform high resolution around it, can the authors supply some supplemental figures illustrating the rest of the protein represents the high-resolution data.

We have shown the electron density of the product-interacting regions in the RdRP in Supplementary Fig. 4 to complement the original Figs. 1-2 (Figs. 2-3 in the revised version). The RdRP density is quite uniform and does not indicate alternate conformation, and we have added related descriptions in teh results section.

5. Fig 1B has letters A,C, B and F but are not described in the legend.

Symbols used to indicate RdRP motifs have been provided in the revised version.

6. Authors need to give more background on why they focused T114 and S115 in the results as the hurdle residues. They just delve into “these two positions” (line 174)

without much context.

We have added descriptions accordingly.

7. Fig. 3 needs to be explained better. The authors assume everyone is familiar with their system. For example, is it just CTP being added? It isn't but is presented such. Does F indicate the fluorophore? Please add a pipeline or include more details in Fig. or legend.

We have modified panel B accordingly (Fig. 4B in the revised version) and added descriptions in the figure legends to clarify the system.

8. Authors do not provide illustrations of the electron density for the motif G hurdle residues (T114-S115) and the basic residues K127/R188 which make key interactions with the nucleic acids. The hurdle resides (T114-S115) are only shown as atomic spheres in already built models (e.g. Figures 4A, 4D, S1B, S4B). The density of the electrostatic interactions between the K127/R188 side chains and the -1/-2 template phosphates is not shown for any of the intermediates. However, authors show electron density for other residues such as I176, D238, and D329 but do not mention them in the main text. The authors should provide electron density figures for residues T114-S115 and K127/R188 to show how they are modeled and how they are interacting with the RNA.

We have provided the Supplementary Fig. 5 in response to this concern. We have also added descriptions modified panel B accordingly (Fig. 4B in the revised version) and added descriptions in the figure legends. We have also added the description I176, D238, and D329 in the main text.

9. The authors outlined a procedure for their RdRP EC crystallization, which involves prolonged incubation of the RNA scaffold in the presence of EV71 RdRP (Main Text, Page 17, Line 368-375). RNAs are susceptible to degradation especially if the protein component of the complex is contaminated with RNases that are carried over during purification. Degradation of the RNA scaffold would have effects on the observed structural intermediates. What measures did the authors use to minimize RNA degradation (e.g RNase-free purification of EV71 RdRP)? Did should run a gel on

mature EC crystals to see if the RNA scaffold and product RNA are still intact after crystallization.

DEPC-treated water was used wherever possible and practical in RNA preparation, EC assembly, and EC crystallization. We have added related descriptions in the methods section. In the time scanning reverse translocation soaking trials, the starting structure and the ending structure both have the template RNA resolved with full occupancy, and the alterative-conformation intermediate structure was obtained with a shorter soaking period than that of the ending structure (2 min vs. 10 min as mentioned in the legend of the Supplementary Fig. 2). Therefore, either the EC upon crystallization or the EC during soaking trials is less likely subjected to degradation.

10. The main structural focus of the paper is the nucleic acids near the site of catalysis. RNA-RNA (template-product) and nucleic acid/protein interactions with neighboring RdRP motifs are compared and highlighted between the translocation intermediates. However, the authors later state that 'the goal of translocation is to “move” the entire polymerase' (Main Text, Page 11, Line 241) and that the 'conformational changes in translocation are not expected to be localized around the active site' (Main Text, Page 12, Line 243-244). Could the authors elaborate on any conformational changes that they observe in the overall structure of the EV71 RdRP translocation intermediates? Perhaps, the authors can align each structure and provide RMSD values for the comparisons (e.g. overall RP comparisons and nucleic acid/motifs comparisons)?

For "global" conformational changes upon translocation, we actually meant the global movement of RNA relative to polymerase. There is no large-scale conformational change occurring during translocation. We have modified our descriptions accordingly and the new Supplementary Fig. 5 also illustrates the most prominent but local conformational changes in around the RdRP active site.

11. In the S6M structure, the authors show a loss of interactions between K127/R188 and the -1/-2 template phosphates that are usually maintained in the forward (S6A and S6B) and reverse (S6RA and S6RB) translocation intermediates. Authors should expand on the conservation and the roles of the K127/R188 residues. Have the

authors performed mutational studies on these residues to assess their roles in catalysis or translocation?

Based on the structural observation, we agree that it is worth testing the impact of these two residues on RdRP catalysis. As these two residues in not even highly conserved in the *Picornaviridae*, we would consider testing their impact in future studies with specific focus on enterovirus RdRP function, similar to our judgment on the H113 testing in response to reviewer 1 and the G117 testing below.

12. The authors capture an intermediate EC state (S6M) by using the T114S-S115T mutant. They proposed the stability of this state is comparable to pre- and post-translocated states (Main Text, Page 11, Line 221-223). To test this, they soaked CTP into the crystal to induce forward translocation (C2) and found that they arrived at the same intermediate state as C0 (absence of CTP) (Main Text, Page 11, Line 223-229). The authors should look at reverse translocation with the S6M intermediate by using PPi and divalent metal ions (similar to what they did to obtain S6RA and S6RB). This would provide evidence that the observed intermediate seen with mutant is between the pathway from pre- to post-translocation (Figure 5C). This is not necessary for this paper, just a friendly suggestion.

We thank this reviewer for this suggestion.

Minor comments

1. Main Text, Page 2, Line 14. Change 'that similar strategy' to 'that a similar strategy'.

Corrected.

2. Main Text, Page 2, Line 9. Change ‘regular' to forward'

We have replace all "" regular translocation with forward translocation in the manuscript. Corrected.

3. Main Text, Page 3, Line 32. Change 'the understands of the' to 'insights into'.

Modified as suggested.

4. Main Text, Page 5, Line 91. Change 'since equivalent' to 'since the equivalent'.

Modified as suggested.

5. Main Text, Page 6, Line 117. Change 'this type of intermediates' to 'these types of intermediates'.

We have used "these" to replace "this type of ".

6. Main Text, Page 10-11, Line 216-217. The authors should define what the 'regular conformation' is and how the -1/-2 template phosphates in the intermediate 'switch' to this conformation. In Figure 4D, the -1/-2 phosphate positions are very different between S5 and S6M.

We have added Supplementary Fig. 5 and provided detailed information to differentiate "regular" and "unusual" backbone conformations. Eye-catching arrows are used in this figure to aid the visualization of RNA movement upon translocation.

7. Main Text, Page 11, Line 234. Change 'the 10s-100s-ncleotide per second rapid synthesis in a processive manner' to 'rapid synthesis in a processive manner (tens to hundreds of nucleotides per second)'

Modified as suggested.

8. Main Text, Page 11, Line 240. Remove 'as an entity'.

Modified as suggested.

9. Main Text, Page 12, Line 261. Change 'intermediates for easily' to 'intermediates from easily'.

Modified as suggested.

10. Main Text, Page 13, Line 279-280. Change 'rate-limiting step with high energy barrier comparable to that in the regular translocation may not exist' to 'the rate-limiting step present in regular translocation may not exist'.

Modified as suggested.

11. Main Text, Page 25, Figure 3A. Could the authors comment on the conservation of G117 in the motif G? Did they also mutate this residue in their enzymological studies?

We thank this reviewer for pointing out the conservation of glycine in motif G. G117 or its equivalents in other RdRPs is not interacting with the RNA template. For many but not all RdRPs from positive strand or ds RNA viruses, the EV71 RdRP G117 equivalent position is at the starting residue of a 4-residue turn. The conservation of the glycine is therefore possibly related to the folding requirement of motif G by providing a flexible backbone, likely only playing a structural role. We have commented on this in the results section. We have not tested G117 mutations in the enzymological studies and may be interested in

testing it with above-mentioned H113 and K127/R188 in the future.

12. Supplementary Information, Page 3, Figure S2 (bottom-right). C2S4/5 should be labeled with '(Mn)' since it was generated using PPi and MnCl2.

Modified as suggested.

13. Supplementary Information, Page 4, Figure S3, Class V. 'amplitue' should be corrected to 'amplitude'.

We actually used "amplitude" in the original version and have double-checked the revised version.

14. Supplementary Information, Page 5, Figure S4. Show electron density for the C2S6M map similar to Figures 1B, 2B, 4A, S2.

Composite SA omit electron density has been shown for this figure (now Supplementary Fig. 6).

15. Supplementary Information, Page 7, Supplementary Video Legends. Provide timestamps for each structure shown in the video. For clarity, authors should label the states and key residues and bases in Video S1 and Video S2.

We have modified our video legends to indicate the equal duration of the three segments in the Supplementary Video one, the coloring scheme of the +1 template phosphate and critical RdRP residues to aid the visualization.
